# Predictability of cortico-cortical connections in the mammalian brain

**DOI:** 10.1162/netn_a_00345

**Published:** 2024-04-01

**Authors:** Ferenc Molnár, Szabolcs Horvát, Ana R. Ribeiro Gomes, Jorge Martinez Armas, Botond Molnár, Mária Ercsey-Ravasz, Kenneth Knoblauch, Henry Kennedy, Zoltan Toroczkai

**Affiliations:** Department of Physics, University of Notre Dame, Notre Dame, IN, USA; Center for Systems Biology Dresden, Dresden, Germany; Max Planck Institute for Cell Biology and Genetics, Dresden, Germany; Max Planck Institute for the Physics of Complex Systems, Dresden, Germany; Univ Lyon, Université Claude Bernard Lyon 1, INSERM, Stem Cell and Brain Research Institute, Bron, France; Department of Computer Science, Reykjavik University, Reykjavík, Iceland; Faculty of Mathematics and Computer Science, Babeş-Bolyai University, Cluj-Napoca, Romania; Faculty of Physics, Babeş-Bolyai University, Cluj-Napoca, Romania; Transylvanian Institute of Neuroscience, Cluj-Napoca, Romania; National Centre for Optics, Vision and Eye Care, Faculty of Health and Social Sciences, University of South-Eastern Norway, Kongsberg, Norway; Institute of Neuroscience, Center for Excellence in Brain Science and Intelligence Technology, Chinese Academy of Sciences, Shanghai, China; Shanghai Center for Brain Science and Brain-Inspired Intelligence Technology, Shanghai, China

**Keywords:** Machine learning, Neuroanatomy, Neocortex, Primate, Rodent

## Abstract

Despite a five order of magnitude range in size, the brains of mammals share many anatomical and functional characteristics that translate into cortical network commonalities. Here we develop a machine learning framework to quantify the degree of predictability of the weighted interareal cortical matrix. Partial network connectivity data were obtained with retrograde tract-tracing experiments generated with a consistent methodology, supplemented by projection length measurements in a nonhuman primate (macaque) and a rodent (mouse). We show that there is a significant level of predictability embedded in the interareal cortical networks of both species. At the binary level, links are predictable with an area under the ROC curve of at least 0.8 for the macaque. Weighted medium and strong links are predictable with an 85%–90% accuracy (mouse) and 70%–80% (macaque), whereas weak links are not predictable in either species. These observations reinforce earlier observations that the formation and evolution of the cortical network at the mesoscale is, to a large extent, rule based. Using the methodology presented here, we performed imputations on all area pairs, generating samples for the complete interareal network in both species. These are necessary for comparative studies of the connectome with minimal bias, both within and across species.

## Author Summary

Revealed by tract-tracing datasets, communication between the functional areas of the cortex operates via a complex, dense, and weighted network of physical connections with little apparent regularity. Although there are studies showing the existence of nonrandom topological features, their extent has not been clear. Employing a machine learning–based approach, which efficiently extracts structural models from such datasets, here we show that there is a significant amount of regularity embedded in the mammalian connectome. This regularity allows predicting interareal connections and their weights with good accuracy and can be used to infer properties of experimentally untested connections. The structural models are well learned even with small training sets, without overfitting, suggesting the existence of a low-dimensional, universal mechanism for mesoscale cortical network formation and evolution.

## INTRODUCTION

Information in the brain is encoded via the temporal patterns of signals generated by a network of distributed neuronal assemblies (Hebb, 1949; McCulloch & Pitts, 1943), whose organization has been shown to be strongly determined by its weighted connectivity and spatial embedding (Knoblauch et al., 2016; Markov et al., 2013b). This contrasts with technological information networks, where information—including the destination address—is encoded into packets and routed via switches, and where the network structure serves principally as a propagation backbone. In comparison, the structure of brain networks—the connectome (Sporns et al., 2005)—forms an integral part of the processing algorithm itself. It is expected that disruptions to the optimal structure of the connectome will lead to severe neurological deficits (neurodegenerative diseases) even though the network remains connected, as for example, in patients with syndromic autism spectrum disorder (such as with tuberous sclerosis complex), where there is decreased long-range connectivity and short-range overconnectivity, with reduced functional specialization (Peters et al., 2013). In contrast, while long-range connectivity in packet-switching technological networks helps with efficiency of information transfer, it is not necessary (e.g., ad hoc wireless mobile radio networks), as long as there is a path from source to destination for the packets to travel along.

Despite being fundamental for understanding the brain in health and disease, there is limited knowledge of cortical circuitry, which at the microscale is presently intractable, due to the staggering size of its numbers of nodes (neurons) and connections (Frégnac & Bathellier, 2015). What is tractable with current technology, however, is the investigation of the mesoscale, interareal connectivity patterns corresponding to the physical pathways between functionally defined areas, addressed in ongoing electrophysiology and whole-brain imaging efforts to understand cognitive functions (Mesulam, 2012). Note, complete connectomes have been generated in numerous mammalian species using tractography based on diffusion MRI (Assaf et al., 2020; Suarez et al., 2022) and used for comparative network studies (Goulas et al., 2019; Griffa et al., 2022; Mars et al., 2018; Warrington et al., 2022). However, in dMRI tractography the weak signal-to-noise ratio, the limited resolution and abundant false positives lead to only a modest performance in terms of measuring with precision the point-to-point physical connectivity between cortical areas (Donahue et al., 2016). Here, we restrict ourselves to accurate interareal network data inferred from retrograde tract-tracing methods (see below). While the full interareal network (FIN), as determined via tract-tracing is currently unavailable for any mammal, it is obtainable in the foreseeable future, although, requiring highly specialized laboratories.

Among the empirical approaches, retrograde tract-tracing, has emerged as a reliable, high-resolution method to trace neuronal pathways (Köbbert et al., 2000; Lanciego & Wouterlood, 2011). Compared to anterograde techniques, the major advantage of retrograde tract-tracing is that counts of labeled cells provide a reliable metric of connection strength, yielding a weighted, directed and spatially embedded, physical network of connections between brain areas (Gămănuţ et al., 2018; Majka et al., 2020; Markov et al., 2014; Zingg et al., 2014). In these experiments, a site in a single area, referred to as the target area, is injected with a tracer, which then back labels the cell bodies of neurons with terminals ending at the injection site in that target area. Areas external to the target area housing labeled neurons are called source areas. The weight of an interareal connection from source area *j* to target area *i*, defined via the counts of labeled neurons, is recorded as the fraction of labeled neurons *FLN*_*ij*_ found in area *j* (*j* ≠ *i*), when injecting into area *i* (Markov et al., 2011).

Although existing retrograde tracing datasets do not provide the FIN, they do provide edge-complete subgraphs, that is, networks formed by a subset of vertices whose connectivity within this subset is fully known. These studies have shown that interareal cortical networks (Ercsey-Ravasz et al., 2013; Gămănuţ et al., 2018; Horvát et al., 2016; Theodoni et al., 2020) are in a class of their own when compared to other real-world complex networks, including technological information networks. One key distinguishing feature is their high density of binary connectivity (connections existing or not), that is, they contain a large fraction of the maximum number of possible connections: 0.66 for the macaque (Markov et al., 2011) and 0.97 for the mouse (Gămănuţ et al., 2018). At such high-density values, binary connectivity gives little insight and instead, a network’s specificity is largely encoded in the profiles of connection weights of individual areas (Gămănuţ et al., 2018; Markov et al., 2014), which are reflective of the area’s specialization (Bressler, 2004; Bressler & Menon, 2010; Markov et al., 2011; Passingham et al., 2002).

Studies of self-consistent tract-tracing datasets (Horvát et al., 2016; Kennedy et al., 2013) have revealed in both mouse and monkey the action of the so-called exponential distance rule (EDR), which significantly constrains the structure of the interareal networks (Ercsey-Ravasz et al., 2013; Horvát et al., 2016; Markov et al., 2013b; Theodoni et al., 2020). The EDR expresses the empirical observation that axonal connection probability decays exponentially with projection length (Ahn et al., 2006), *p*(*l*) ∼ *e*^−*λl*^, where *λ* = 1/〈*l*〉 is the inverse of the average axonal projection length ($\lambda_{\text{exp}}^{\text{mac}}$ = 0.19 mm^−1^, $\lambda_{\text{exp}}^{\text{mus}}$ = 0.78 mm^−1^). A one-parameter (*λ*), maximum entropy principle–based generative EDR model captures many features of the interareal network in both species, including the frequency distribution of three-node motifs, efficiencies, core-periphery structures, eigenvalue distributions connection similarity profiles, and wire minimization (Ercsey-Ravasz et al., 2013; Horvát et al., 2016; Song et al., 2014; Theodoni et al., 2020). Earlier studies of network measures/quantities in the cortical connectome, such as small-world properties, rich-club, hierarchical modularity, motifs, and so forth, have shown that they deviate significantly from their counterparts in random or randomized networks (used as null models), indicating the existence of nontrivial topological features (Bassett & Bullmore, 2017; Betzel et al., 2018; Meunier et al., 2010; Tononi et al., 1998; van den Heuvel et al., 2012; van den Heuvel & Sporns, 2013). The discovery of the EDR and the fact that it captures most of these network measures with good accuracy, further strengthens the conclusion that the cortical connectome is indeed rule based.

Interareal networks are the evolutionary consequences of genetic prespecification and interactions with the environment (Buckner & Krienen, 2013). Although there is network variability between individuals (Gămănuţ et al., 2018; Markov et al., 2014), there are common features within and across species (Goulas et al., 2019; Horvát et al., 2016; Margulies et al., 2016; Markov et al., 2013b; Mota et al., 2019). This is supported, for instance, by the cross-species consistency of the EDR (Horvát et al., 2016; Theodoni et al., 2020) and of the topographical ordering of areas on the cortical mantle (Krubitzer, 2009). We refer to these generic features as *architectural network invariants*, which we argue, imply predictability of networks. To study this issue, we turn to prediction and machine learning methods and show that they can be used to assess the degree of predictability of brain networks, and thus also usable for predicting missing network data (imputation). Naturally, the accuracy of imputation is determined by the degree of predictability inherent in the data. Moreover, we argue that predictive methods can also be used as *tools* to study structure-function relationships in these networks. Overall, they address the following questions: (i) How well can connections be predicted? (ii) Are certain parts of the network more predictable than others? (iii) How does heterogeneity in predictability relate to cortical function and behavioral features of the species? (iv) How does predictability compare across orders? (v) Can we use predictability as a guide for further investigations?

Two aspects of our approach need to be emphasized. First, the limit to predictability is primarily an inherent property of the data. This is because even the best possible algorithm can extract only so much predictive information, either because the dataset is noisy or the unpredictable part is the result of other variables, independent from those in the dataset, and not contained in it. Although the quality of prediction algorithms can vary wildly, even the best algorithm cannot and should not “predict” information that is not there (e.g., in the case of two pieces of mutually independent data A and B). Secondly, one must avoid overfitting, that is, fitting to noise in the data, as this leads to loss of generalization power and erroneous conclusions.

## RESULTS

### Data Description

**Figure 1.**
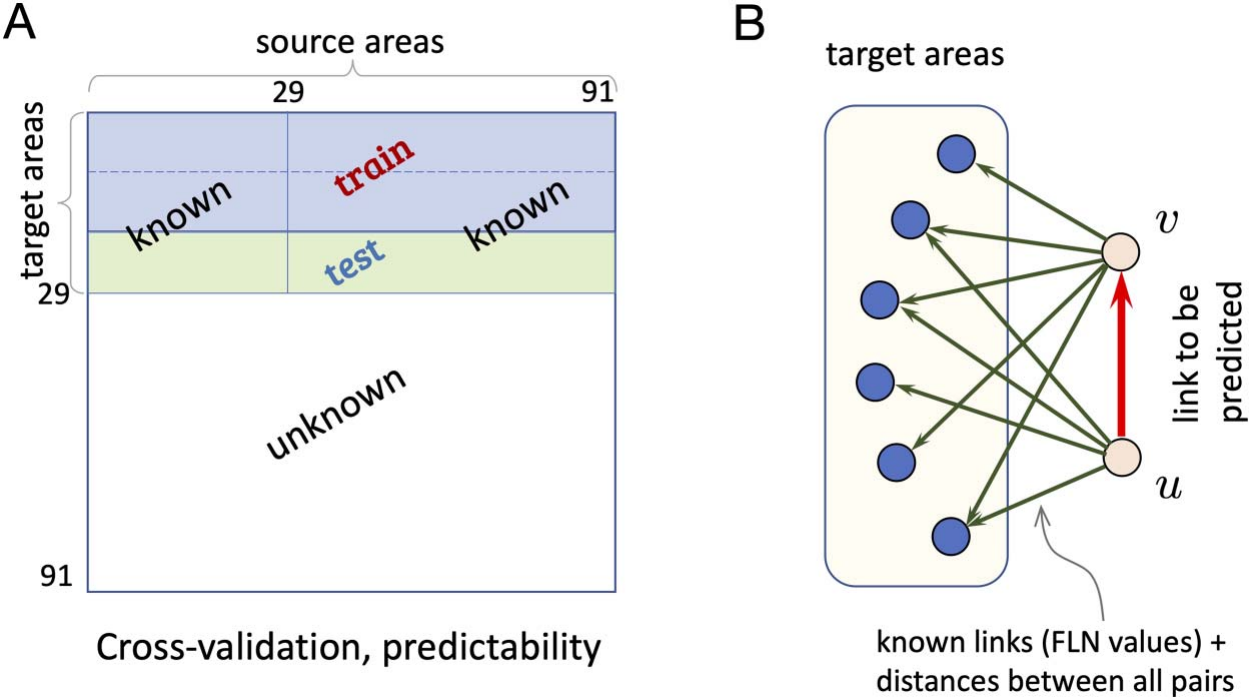
Schematics for link prediction with retrograde tract-tracing data. (A) *k*-fold cross-validation setup for predictability (*k* = 3). (B) Links are predicted based on information (weights, distances) from the out-neighborhoods of its incident vertices.

We rely on two retrograde tract-tracing datasets obtained with consistent methods, one for the macaque (mac) (Markov et al., 2014) and the other for the mouse (mus) (Gămănuţ et al., 2018). The mouse dataset $G_{19\times 47}^{\text{mus}}$ is a matrix of FLN values *FLN*_*ij*_ for 19 injected target areas (*j* is a source, projecting into target *i*) in a 47-area parcellation. The macaque dataset $G_{29\times 91}^{\text{mac}}$ contains the same for 29 injections on a 91-area atlas. Both datasets are provided in the Supporting Information. The full interareal networks (FIN), which are not available for either species, would be the matrices $G_{47\times 47}^{\text{mus}}$ and $G_{91\times 91}^{\text{mac}}$, respectively. Additionally, our datasets contain all pairwise distances along estimated shortest paths avoiding anatomical obstacles, between the area barycenters, recorded in the matrices $D_{47\times 47}^{\text{mus}}$ and $D_{91\times 91}^{\text{mac}}$, respectively (provided in the Supporting Information). Due to the high tracer sensitivity, each injection reveals all the areas that project into the injected target area and thus, the FLN matrix *G*_*T*×*N*_ is a row submatrix of the FIN *G*_*N*×*N*_. Therefore, we either know the full row (corresponding to a target area) or not at all. This is illustrated in Figure 1A where the first *T* rows represent the targets in the full *G*_*N*×*N*_ matrix. The FLN data matrices were preprocessed so that the links have a positive real numeric weight between 0 and 7 (see Methods).

### Link Prediction Framework

Link prediction refers to inferring links from observed network data (Clauset et al., 2008; Liben-Nowell & Kleinberg, 2003; Lü & Zhou, 2011). This can be done at the binary (predicting only if a link exists/1 or not/0) or weighted levels (predicting the associated weight). Binary level predictors are also known as classifiers, whereas weighted predictors are essentially regressors. There are two main families of network prediction methods: Classical Link (CL) predictors and Machine Learning (ML) predictors. CL predictors/classifiers, used extensively in social networks, forecast links at the binary level based on either node neighborhood information (local) or path information (global). This information is formulated into a *predefined model* that generates a score *score*(*u*, *v*) for every ordered node pair (*u*, *v*), which is then used to make the prediction. For this reason, CL methods are susceptible to modeling bias: the models are built using modeler-defined measures as constraints, which might have or not have strong relevance to the features the modeler wants to predict for a given dataset.

ML predictive methods can be used both as classifiers (for binary prediction) or as regressors (for weighted prediction). They predict based on *learning* from samples given a set of features. A feature is a vector of values (feature vector) quantifying what we know about the relationship of a node pair. We train an ML predictor in a supervised fashion (hence they belong to the class of supervised classifiers/regressors, by providing the feature vectors computed for the node pairs in the training set and using the *ground truth* data about the pairs’ connectivity. The predictor then creates a model *autonomously* that best fits the given training set with the given feature vectors, which is then tested against the ground truth in the test data and the classifier’s performance is evaluated. Thus, the fundamental difference between CL and ML is that we impose the model in CL, whereas it is learned in ML. While the ML uses a modeling framework, such as weighted feed-forward neuronal network, it is *not* a model of the graph that is being modeled. The framework just has to have *sufficient complexity* and *sufficient flexibility* that allows tuning its internal state *to any information* that it is learned. In particular, for feed-forward neuronal networks, the Universal Approximation Theorem (Cybenko, 1989; Hornik et al., 1989), allows approximating with arbitrary accuracy any function, given sufficient neurons in the hidden layer. This theorem *guarantees* the absence of modeling bias, given a sufficiently large size of the hidden layer. However, not all ML frameworks have mathematical proofs for them being universal approximators, but they do have both the flexibility and sufficient tunability to work as universal approximators, at least in practice. It is also important to note that given several ML algorithms with such universal approximation properties, they should all have similar predictability performance, given that they truly minimize bias, when learning, which can also be seen in our results below. One way to test that a given ML predictor has extracted the relevant, generalizable information is via the analysis of prediction residuals (discussed below). The residuals should show the characteristics of uncorrelated noise once all the information has been extracted in an unbiased manner.

An important note is that for both CL and ML, the information on which the prediction is based (scores and feature vectors) has to be computable *for all pairs in an identical fashion*, limiting the types of predictors that can be used. In particular, this excludes path-based predictor models (PageRank, Katz, Shortest Path), because there are no paths into some of the vertices of the links to be predicted (the noninjected areas). For both CL and ML, we must use information on outgoing links, these being the only type of information commonly available to all node pairs (see Figure 1B).

The performance of both classifiers (CL, ML) and regressors (ML) is evaluated using cross-validation, which separates the data with ground truth value into two sets: a training set and a test set. The former is used as input information for the predictor, which based on that makes predictions for links in the test set, which is then compared to the ground truth. Here we use *k*-fold cross-validation, which splits the data into *k* equal parts, using in one iteration one of the parts for the test set and the other *k* − 1 parts for training, then this is repeated for every combination of test/training split. Performance metrics are then averaged. To avoid correlations with any predetermined ordering of the input data we randomize the ordering of the target areas in the FLN matrices^1^ before splitting it into *k* parts. We apply *k*-fold cross-validation over multiple randomizations of the target areas, then compute the corresponding averages over all these randomized realizations and all the folds within. For classifiers we use the standard receiver operating characteristic (ROC) curve and the corresponding single scalar metric, the area under the ROC curve (AUC), as performance metrics. The ROC shows the true positive rate TPR = TP/(TP + FN) plotted against the false positive rate FPR = FP/(FP + TN), obtained by moving the threshold value that distinguishes positive and negative predictions. Here TP, TN, FP, and FN are the number of true positive, true negative, false positive, and false negative predictions, respectively. A perfect classifier has 100% TPR and 0% FPR and the ROC curve fills the top left corner of the unit square; a random predictor has 50% TPR and 50% FPR with the ROC following the main diagonal of the unit square; anything below the main diagonal implies an invalid predictor. The ROC curve also has a specific point that corresponds to the maximum prediction accuracy. Accuracy (ACC) is defined as the number of correctly predicted links and nonlinks divided by the number of all predictions, ACC = (TP + TN) / (TP + TN + FP + FN). This point is determined numerically for each ROC curve, and this threshold is used to make the binary predictions during cross-validation. For weighted predictors there are no ROC curves. Instead, we use the mean absolute error (MAE) or the relative MAE (RMAE) between predicted and actual links weights (using RMSE, i.e., root-mean-square error gives very similar results).

Cross-validation helps to quantify not only how well a particular algorithm predicts the presence or absence of links but also to quantify the degree of predictability in the data. Note, the imputation task is only meaningful if the cross-validation results indicate significant predictability in the data. Here we present predictability results (cross-validation) in both species using both CL and ML algorithms at binary and weighted levels. Details of link imputation will be presented in a subsequent publication.

### Network Predictability in the Macaque and Mouse

#### Binary link prediction.

The scores *score* (*u*, *v*) generated by the CL algorithm for every node pair (*u*, *v*) are based on formulas that express various types of network information. These formulas, used typically in social networks, provide summations over nodes with incoming links from both *u* and *v*. Since retrograde tracing data only reveal the incoming links to the target areas, the predictor formulas must be modified accordingly (see Materials and Methods). In the case of ML classifiers, we need to specify the feature vectors.

**Figure 2.**
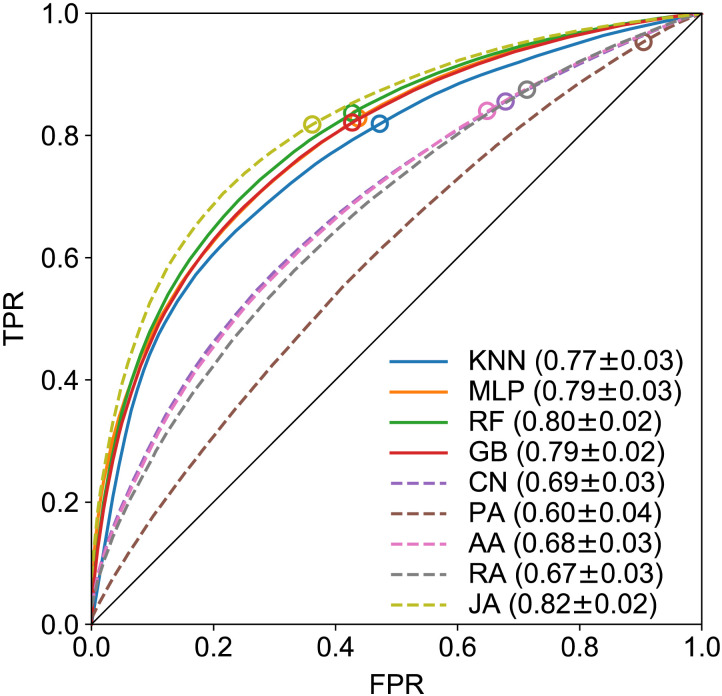
ROC curves for binary link prediction in the macaque. Dashed lines are from CL predictors: CN = common neighbors, PA = preferential attachment, AA = Adamic-Adar, RA = resource allocation, JA = Jaccard index. The continuous lines are from the four best ML classifiers, based on the full FLN-plus-distance feature vectors: KNN = *k*-nearest neighbors, MLP = multilayer perceptron, RF = random forest, GB = gradient boosting, using *k*-fold cross-validation, with *k* = 3. The markers indicate the location of the maximum accuracy thresholds.

Figure 2 shows the macaque ROC curves for four ML classifiers (solid lines) based on full information, that is, feature vectors composed of both FLN values and distances (see Table 3) for details. We have tested several combinations of data for feature vectors and found the results to be inferior to those based on full information (Supporting Information Figures S1–S5). We also tested other classifiers, including DecisionTree, AdaBoost, and NaïveBayes, but they performed worse than those shown here. It is clear that with the exception of JA (modified Jaccard), the CL predictors do not perform as well as the four ML classifiers. The ML classifiers were tested against overfitting (Supporting Information Figures S6 and S7 show the case of the MLP). They were also tested using different *k* values for the number of folds (Supporting Information Figure S8). The approximately 80% AUC obtained consistently by the top performing classifiers indicates high predictability embedded in the macaque interareal network, suggesting the existence of architectural invariants and corresponding mechanisms (Figure 2). This analysis cannot be applied to the mouse dataset, (see the ROC curves in the Supporting Information Figure S9) due to its ultra-high connectivity density of 97%, which causes a strong bias (because the classifiers have only 3% true negatives to learn from). This implies that only weighted predictions can be made in the mouse brain, as presented in the next section.

**Figure 3.**
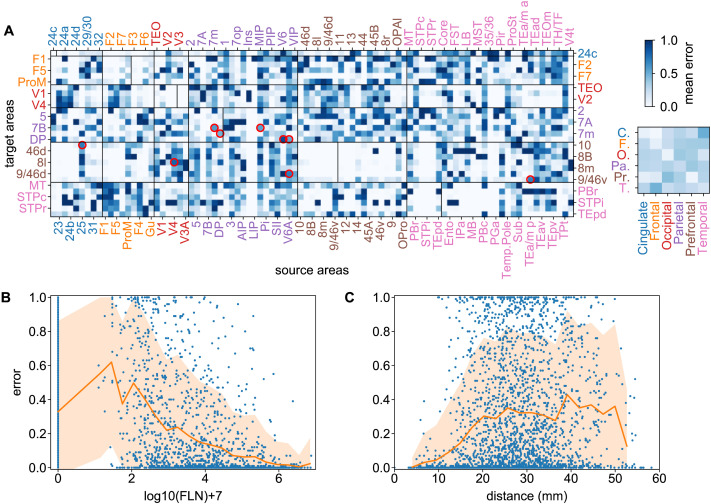
Binary prediction heterogeneity in the macaque brain. (A) Prediction error matrix for all known links (3-fold cross-validation) generated by gradient boosting (GB). Vertical lines within the main diagonal boxes, separate targets (to the left of the line) from noninjected areas (to the right of the line). Red circles indicate strong links (with weights > 5) with high prediction errors (*ϵ* > 0.5). Along with their weights *w* and their errors *ϵ*, these are: V6 → DP (*w* = 5.3, *ϵ* = 0.80), V4 → 8l (*w* = 5.1, *ϵ* = 0.72), 25 → 10 (*w* = 5.2, *ϵ* = 0.66), *V*6*A* → 9/46d (*w* = 5.3, *ϵ* = 0.65), TEa/mp → 9/46v (*w* = 5.3, *ϵ* = 0.65), MIP → 7B (*w* = 5.9, *ϵ* = 0.58), 7m → 7B (*w* = 5.3, *ϵ* = 0.57), DP → 7m (*w* = 5.1, *ϵ* = 0.52) and V6A → DP (*w* = 5.6, *ϵ* = 0.52). Inset matrix shows interregional errors obtained by averaging errors within submatrices corresponding to cortical lobes. (B) Prediction errors as function of link weights and (C) as function of link projection distance. The vertical line in panel B at 0 are all the node pairs for which the prediction was *nonlink*, while panel C contains all *links* and all *nonlinks*. The orange shaded areas in B and C represent one standard deviation from the average (orange line). The definition of error measure is given in the main text. Area abbreviations with corresponding area names and region assignments are provided in the Supporting Information Table S1.

Figure 3 shows individual link prediction errors in the macaque data for all the links with a corresponding ground truth value (lighter colors correspond to smaller errors). A prediction (link existing/1 or not/0) was obtained for every *k*-fold run in all area pairs *i*, averaged over 100 randomized *k*-fold run predictions, generating a prediction 〈*y*_pred_(*i*)〉. The error is calculated via *error*(*i*) = |*y*_true_(*i*) − 〈*y*_pred_(*i*)〉|, where *y*_true_(*i*) ∈ {0, 1} is the true binary link value.

**Figure 4.**
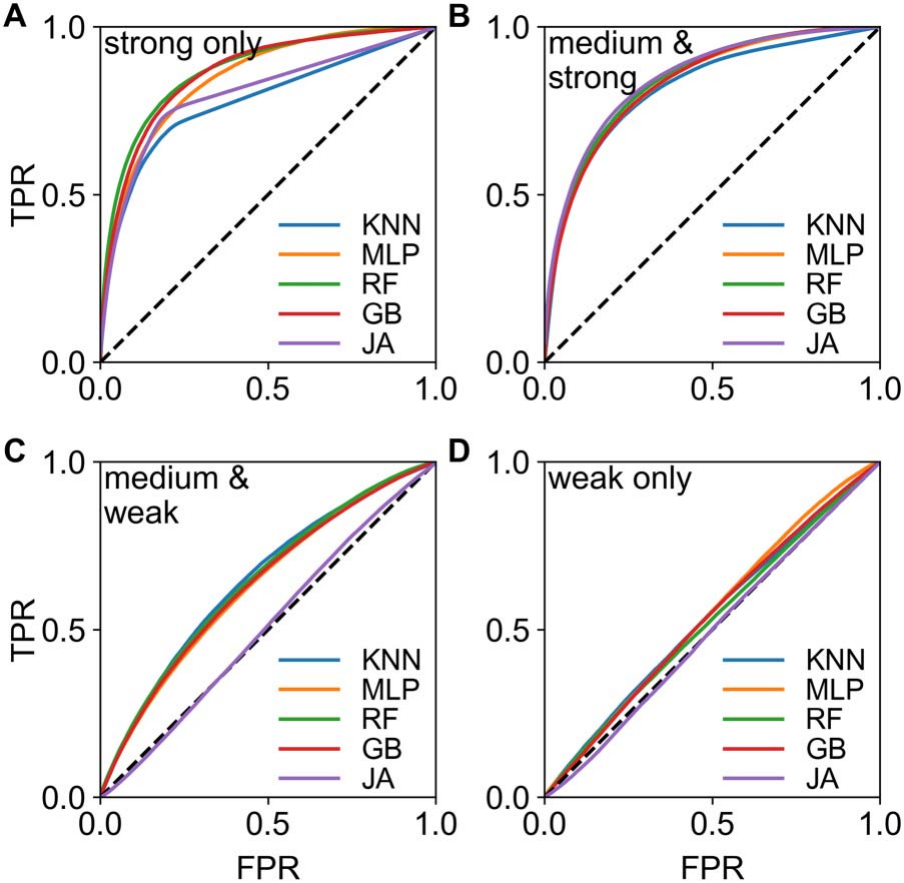
Binary predictability within link weight classes in the macaque. Predictability within only (A) Strong links *w*_*ij*_ ≥ 5 (359 links), (B) Strong-&-Medium *w*_*ij*_ ≥ 3 (1,164 links), (C) Medium-&-Weak *w*_*ij*_ ≤ 5 (2,251 links), and (D) Weak links *w*_*ij*_ ≤ 3 (1,446 links). The AUC values and errors in panel A: KNN (0.65 ± 0.02), MLP (0.75 ± 0.04), RF (0.75 ± 0.03), GB (0.72 ± 0.03), JA (0.69 ± 0.03); in B: KNN (0.78 ± 0.03), MLP (0.81 ± 0.04), RF (0.81 ± 0.03), GB (0.81 ± 0.03), JA (0.83 ± 0.02); in C: KNN (0.65 ± 0.03), MLP (0.63 ± 0.05), RF (0.65 ± 0.03), GB (0.63 ± 0.03), JA (0.59 ± 0.04); in D: KNN (0.45 ± 0.03), MLP (0.50 ± 0.06), RF (0.46 ± 0.03), GB (0.47 ± 0.03), JA (0.55 ± 0.02).

The inset in Figure 3A is a matrix of link prediction error heterogeneity by cortical brain regions. This shows that links from the frontal to temporal regions are less predictable (bottom row, second column), while links from frontal to cingulate (and prefrontal) are more predictable, and so forth. In addition, links within functional regions are more predictable than between regions (main diagonal of the small matrix). This suggests that predictability is possibly distance and thus weight dependent, since from EDR, we know that short/long connections are preponderantly strong/weak. Figure 3B and C show how prediction errors behave as a function of link weights and distance, demonstrating the action of a distance rule on predictability. In order to disentangle the effects of distance/weight, we examined predictions based only on links of certain strengths: Strong, *w*_*ij*_ ≥ 5; Medium-&-Strong, *w*_*ij*_ ≥ 3; Medium-&-Weak, *w*_*ij*_ ≤ 5, and Weak, *w*_*ij*_ ≤ 3. In one analysis, we consider the data only within one weight class and measure the predictability within that class. This is presented in Figure 4, clearly showing that weak links are not predictable at the binary level (panel D), that is, the weak (mostly long-range) links carry no information about each other. This is a significant observation that we revisit below, in our weighted prediction analysis. The maximum binary predictability is within the Strong-&-Medium group. The somewhat weaker predictability of the Strong group is possibly due to it being the smallest and the existence of some strong links with high unpredictability (red circles in Figure 3A) within this group, note V4 → 8l is part of a strong loop (Markov et al., 2013b, 2013c; Vezoli et al., 2021).

We obtain the same conclusion if, after training the models within one weight class only, we predict all links in the test set, irrespective of their ground truth weight class, then decompose the predictions by ground truth weight classes (see Supporting Information Figure S10).

#### Weighted link prediction and comparisons between mouse and macaque.

In order to predict link weights, we turn to supervised regression methods. This excludes CL algorithms as they are designed uniquely for binary link predictions. Since all our ML classifiers are available as regression algorithms as well, they can be used for weighted link prediction. The same feature vectors are used but the ground truth now is the actual link weight, *w*_true_. To evaluate the performance and the amount of predictability inherent in the network we employ the same *k*-fold cross-validation scheme, but the performance metric has to be modified (there are no ROC curves in weighted link prediction). One could use the mean absolute error (MAE) obtained as the absolute value of the difference between the predicted and the actual weight |Δ*w*| = |*w*_pred_ − *w*_true_|, averaged over the 100 *k*-fold predictions; however, since FLN values vary over orders of magnitude, the MAE of a weak link is not easily comparable to that of a strong link. To take this into account, we use the relative MAE (RMAE), which is the MAE divided by the ground truth strength of the predicted link, |Δ*w*|/*w*_true_. Thus, the RMAE value is the fraction of the link weight that is not predicted. For example, an RMAE of 0.2 means that 80% of the link weight *w* was predicted and 20% was not. An RMAE of 2 reflects an error of 200% compared to the true link strength. Similar to binary prediction, when comparing the performance of several classifiers, GB, KNN, MLP, RF (see Materials and Methods for abbreviations) emerge as the four top predictors.

**Figure 5.**
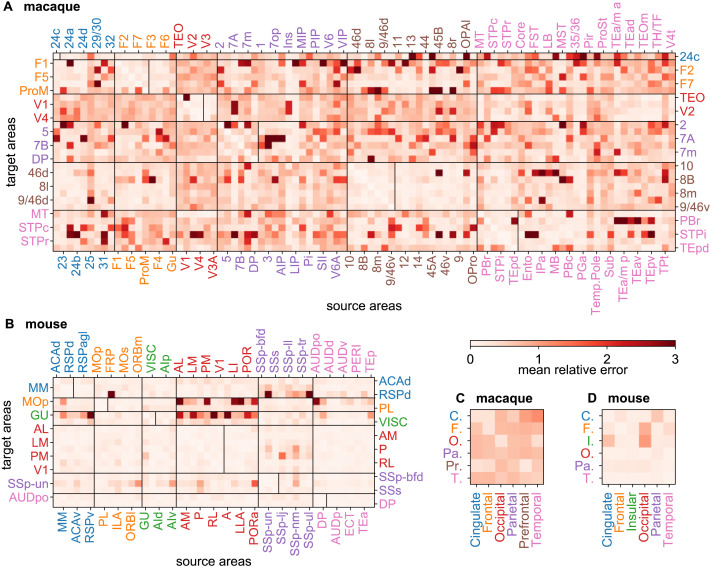
Prediction error heterogeneity for link weights. (A) Weight prediction error (defined as relative mean absolute error, RMAE) matrix for all known links with 3-fold cross-validation, in the macaque, generated by GB and (B) in the mouse. The vertical lines within the main diagonal boxes, separate targets (to the left of the line) from noninjected areas (to the right of the line). (C) interregional error matrix for the macaque (averaged from the matrix in A) and (D) for the mouse (averaged from the matrix in B). For nonlinks, the RMAE was calculated using the lowest statistically acceptable FLN value of 8 × 10^−7^ for the ground truth value (corresponding to a weight of *w* = 0.9). Area abbreviations with corresponding area names and region assignments are provided in the Supporting Information Table S2.

Regressors work by minimizing a cost function (such as the root-mean-square error RMSE) over the training set, when finding the best fitting model, which in turn is used to predict the test set. Analysis of prediction residuals provides both an efficient test of the capacity of the predictor to capture the signal part of the data as well as a means of ranking performance. This analysis shows that GB performs somewhat better compared to RF, MLP, or KNN. Supporting Information Figure S11 shows the results from the analysis of the prediction residuals for the GB algorithm. A featureless scatter plot of the residuals versus predicted values, as shown in Supporting Information Figure S11C, indicates that the signal portion of the data has been well learned by the predictor. For simplicity, in the following we show predictions based only on GB. Figure 5A and B show the prediction error (RMAE) matrices for both the macaque and mouse. Note the strong similarity of the patterns between Figure 5A and Figure 3A for the macaque. At the weighted level as well, some links are more predictable than others. The matrices at the regional level (Figure 5C and D) also show heterogeneity: for example, across species, temporal to occipital is highly predictable, whereas occipital to frontal is less so. Globally, the mouse network appears more predictable than the macaque (overall lighter matrices for the mouse).

**Figure 6.**
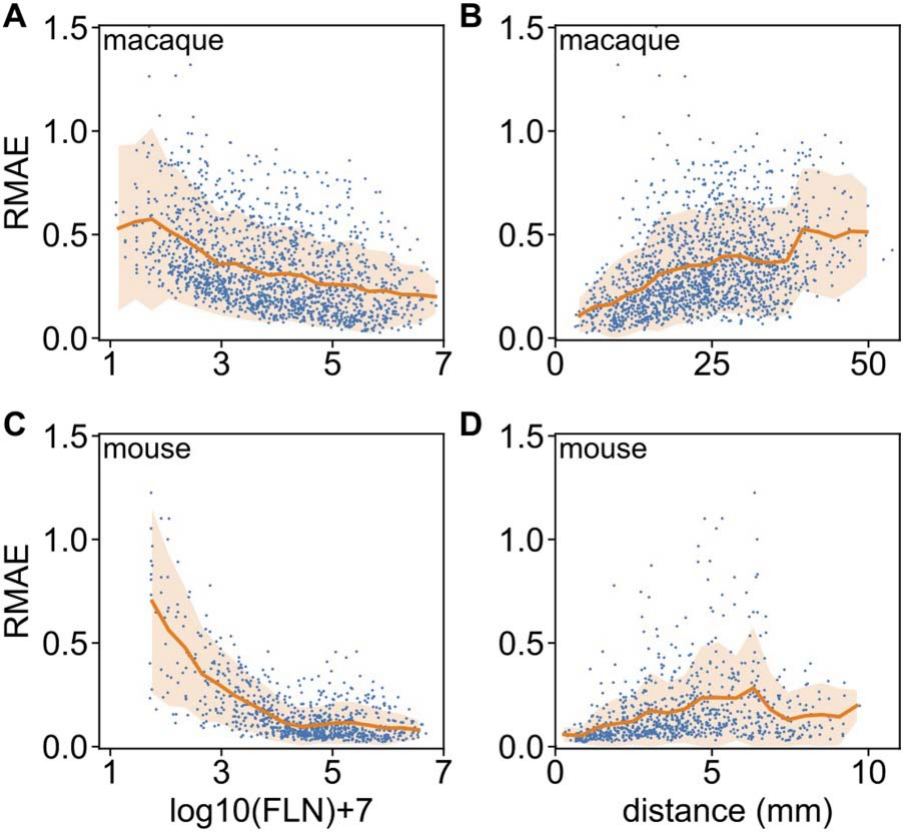
Weighted prediction errors as function of link strength and distance, using the prediction data from Figure 5. (A) Relative mean absolute error RMAE versus link weight and (B) versus projection distance in the macaque for every predicted link. (C) Same as panel A, and (D) same as panel B, for the mouse. The continuous line is the mean value, the orange shaded area corresponds to one standard deviation. Panels do not contain data for no connections.

This is further demonstrated in Figure 6, where we plot RMAE values as function of link weight and as function of link projection lengths (distance). While in both species weaker links are harder to predict, comparing Figure 6A to, C we see that the medium-to-strong links are much more predictable in the mouse than in the macaque, but the situation is reversed for the weakest links. Similarly, long-range links are harder to predict in both species than shorter ones. Overall, weighted links are more predictable in the mouse than in macaque.

We quantify link predictability globally, and by weight classes in Table 1. Predictions (3-fold cross-validation) were made on the full dataset (including links with nonzero weight and also nonlinks) using the GB algorithm and errors computed and averaged within the corresponding groups. The RMAE values in Table 1 show that unlike stronger links, weak links are not well predicted in either species. The stronger links are in general 2-fold more predictable in the mouse than in the macaque. The nonlinks, however, are better predicted in the macaque, likely due to the fact that there are only 3% nonlinks in the mouse dataset. Since the larger errors are associated with the nonlinks, we performed the predictability analysis also on a reduced dataset, with only actual links included (nonlinks excluded) (see Supporting Information Table S3). Except for weak links, predictability improved in general, with mouse links being 1.5 times more predictable than the macaque ones.

**Table 1.** Prediction errors by link weight

**Nonlinks included**	**Macaque**		**Mouse**		**Mac/Mus**
	MAE	RMAE	MAE	RMAE	RMAE ratio
Weak (*w*_cut_ < *w* < 3)	1.081	0.460	1.032	0.446	1.033
Weak-&-Medium (*w*_cut_ < *w* < 5)	1.173	0.365	0.647	0.196	1.862
Medium-&-Strong (*w* > 3)	1.255	0.288	0.565	0.127	2.274
Strong (*w* > 5)	1.324	0.237	0.569	0.102	2.313
All links (*w* > *w*_cut_)	1.207	0.336	0.622	0.166	2.025
Nonlinks (*w* ≤ *w*_cut_)	1.498	1.101	2.911	2.288	0.481
Both links and nonlinks	1.318	0.628	0.683	0.222	2.829

*Note*. MAE = mean absolute error |Δ*w*| = |*w*_pred_ − *w*_true_|, RMAE = relative mean absolute error |Δ*w*|/*w*_true_. For “nonlinks” only, for the relative error, we used the estimated experimental lower cutoff value of *w*_true_ = *w*_cut_ = 0.9, corresponding to an *FLN* = 8 × 10^−7^.

**Figure 7.**
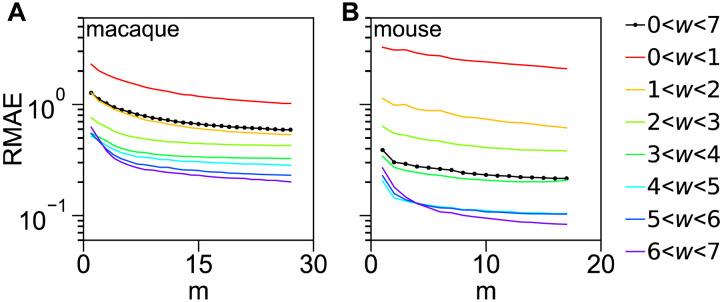
Scaling of prediction errors as function of input data size in a leave-one-out analysis. The relative mean prediction errors RMAE (of weights) are computed for areas internal to a set of *m* targets for both macaque (A) and mouse (B), then plotted as function of *m*; see the Methods section for description. The errors are separated by link weight class. Note the logarithmic scale on the *y*-axis.

Finally, we discuss the issue of scaling of predictability with the amount of data used for training. Here *m* − 1 is the size of the training set; see the Methods section “Scaling and Leave-One-Out Analysis” for definitions and procedure description. Figure 7 shows this scaling as function of input data set size *m*. An interesting conclusion is that the ML predictors learn the structure in the data quickly for the medium-to-strong links, and the improvement after that is relatively modest, although more significant for the weak links (the *y*-axis is on log-scale). See also Supporting Information Figure S12 for another approach, leading to the same conclusion.

## DISCUSSION

Using machine learning methods, we demonstrated that the network of the mammalian cortex contains significant levels of structural predictability, further strengthening previous observations that the formation and evolution of the cortex is rule based. Moreover, our approach allows quantifying the level of predictability in the datasets, which can be used also as a tool for function-structure analyses and interspecies comparisons. Note that the consistent empirical methodology used to generate the two retrograde tract-tracing network datasets in macaque and mouse does allow for interspecies comparisons, using the edge-complete portions of the datasets (Horvát et al., 2016).

At the binary level, predictions on the macaque dataset show that there are significant differences in levels of predictability within the weight classes: while strong and medium links are well predictable, weak links are not. Note that this is solely due to the way the links of different strengths (in the 0–7 weight range) and their lengths are distributed across the network structure. There is no a priori *reason* for the algorithms not to be able to predict weak links (*w* ≅ 1) compared to strong links (*w* ≅ 6), given that it uses simple (0–7) weight values, which are represented as O(1) numbers throughout the network. One can set up artificial networks in which none of the categories are predicted well, or the performance of prediction is flipped (weaker are predicted better and stronger are not). An example for the former is obtained by taking the original data network and rewiring its links, obtaining the configurational model; in this network predictability falls in all categories (see Supporting Information Figure S13B). An example for the flip is shown in Supporting Information Figure S13C in which we use an artificial weight distribution that flips the predictability: the weak and the weak-medium links are much better predicted than the strong. Note that the Jaccard (JA) CL algorithm consistently predicts badly, because it is based on a *preconceived model* that is no longer relevant for this new artificial weight distribution.

Predictions at the level of link weights confirm the same conclusion as at the binary level, but with more details and now also for the mouse. The analysis also shows that overall, compared to macaque, the mouse brain is more predictable. However, the weakest connections in the mouse (compare panels A and C in Figure 6) are less predictable than in macaque, suggesting comparatively less specificity. One argument one could raise regarding the non-predictability of the weak/long-range links in both species is that the experimental data on weak links may be much noisier. However, this is not true, the consistency of the data on weak links has been demonstrated in several analyses (Markov et al., 2011, 2013a, 2014; Gămănuţ et al., 2018).

It is important to note that these predictability measures are all based on the features of link weights and projection distances. Including additional, biologically relevant features such as cell types, transcriptomic specialization, and anatomical gradients would be expected to lead to an improved refinement of the predictability (Burt et al., 2018; Wang, 2020), including for weak/long-range links. See further discussions in the last paragraph.

Link prediction efforts in the context of brain networks are fairly limited, but they go back to a 1998 paper by Jouve et al. (Jouve et al., 1998) using a seminal dataset on the macaque visual system (Felleman & Van Essen, 1991). Follow-up works appear almost a decade later (Cannistraci et al., 2013; Chen et al., 2020; Costa et al., 2007; Hinne et al., 2017; Hoff, 2007; Nepusz et al., 2008; Røge et al., 2017; Shen et al., 2019), but all (including Jouve’s) are based on preconceived network models using summary network statistics whose parameters are fitted to the data, and then used to make predictions on missing links. One problem is that the summary statistics are obtained on incomplete datasets, which bias these statistics, a bias which is then built into the prediction. A further possible bias is that these models are taken from the field of social networks. Here, by comparing the performance of CL link predictors (social science inspired, model-imposed) with machine learning predictors (that learn the structure from the data, without imposing specific models), we have shown that the latter approach achieves significantly better predictions than most of the model-based predictors. The Jaccard coefficient is the only successful CL predictor because its formula happens to correlate with a property of the link weight distributions in the brain, namely the triangle inequality. This holds for spatial networks, a property respected by the link weights of the brain, due to the action of the EDR: if areas A and B are close to each other (strong link) and area C is far from A (weak link), then C will also be far from B (weak link), mimicked by the Jaccard index as well. Although, in general, it is better to have a model-based predictor as it is based on one or more well-defined network properties responsible for good predictability, one usually cannot know a priori what those properties are. It might be the case that it is not a single, simple-to-formulate property, but a collection of complex features, that lead to good predictions. The advantage of the ML predictors is that they learn the features for predictability from the dataset and do make good predictions, almost independently of how complex those features are; the disadvantage, however, is that it is very difficult to “interrogate” ML predictors so as to extract those features in a human-understandable format. However, it is recommended to try starting out with ML predictors as they indicate the level of predictability inherent in the dataset. If there is a significant amount of predictability, then one can start working toward narrowing down the features responsible for it.

Given the amount of link predictability inherent in the datasets for both species, we can now use our ML predictors to impute all the missing connections, thus generating samples for the weighted FIN in both species (91 × 91 for the macaque and 47 × 47 for the mouse). Edge-complete, full connectivity matrices are crucial when studying network/graph theoretical properties since missing links significantly bias summary statistics, such as path-length distributions, centrality measures, and even simpler summary statistics such as degree distributions and network motifs distributions. Samples of FIN for both species have been included in the accompanying data package.

Recall that the EDR model (Ercsey-Ravasz et al., 2013; Horvát et al., 2016; Markov et al., 2013b; Theodoni et al., 2020), mentioned in the introduction, captures many features of the cortical networks in both species. One may ask, what is the amount of predictability in the EDR model, using the same distance matrix as in the data, and the empirical decay rates *λ*? We find that the top predictors achieve a better performance on the EDR model networks (an AUC of 0.86, see Supporting Information Figure S14) than on the experimental connectivity data (an AUC of 0.80; Figure 2). This is expected, given that these networks are, by definition, rule based, with some level of randomness (Ercsey-Ravasz et al., 2013).

Machine learning methods can be used to explore the connectome in several ways. First, as a guide to future neuroanatomical experiments. Prediction analysis could propose optimal injection sites based on levels of expected surprise. Second, prediction analysis can be used to examine the known connectivity. Here, those areas for which predictions *differ* significantly from the observed connections would be of particular interest, or alternatively would prompt reexamination of the empirical data. Cases where large deviations are observed deserve close scrutiny; they could correspond to the appearance of a novel information processing modality, reflecting a significant evolutionary branching event in the history of the species. The fact that long distance and therefore weak connections are systematically unpredictable is intriguing, because anatomically we have shown that they are highly consistent across individuals (Gămănuţ et al., 2018; Markov et al., 2013a), suggesting that such connections could have important functions (Csermely, 2006; Granovetter, 1973), which have been largely missed by numerous studies (Kennedy et al., 2013) and which could have relatively recent evolutionary origins. In particular, our finding that the weak, long-range links are not predictable based on distances and weights alone is consistent with earlier observations that in-link similarity, in terms of shared inputs of two targets, decreases with increasing distance between the two target areas (Horvát et al., 2016; Markov et al., 2013a) both for macaque and also for mouse (Horvát et al., 2016). The in-link similarity index measures the degree to which two target areas receive input or avoid receiving input from common sources. This is consistent with the findings here, namely that compared to macaque there is slightly more predictability of long-range links in the mouse (Figure 5B and D). The globally greater predictability in mouse compared to macaque, could imply a greater degree of gradualism in the evolution of rodents compared to primates (Gould & Eldredge, 1977). When the similarity indices are overlayed between the two species on the same plot, as a function of rescaled distance (by the average interareal distance in the respective species) one finds strong overlap up to medium distances, after which the plots deviate, as shown in Horvát et al. (2016). Another piece of evidence is the comparison of the decay of the probability of extrinsic (to the target area) connections (when injecting into target V1) with rescaled distance (by the average interareal distance) between three species, namely, macaque, mouse, and microcebus, also shown in Horvát et al. (2016). These histograms strongly overlap up to medium distances, clearly following the EDR, after which they separate in their own ways. This is again, consistent with the machine learning observations presented here. Good predictability of the long-range/weaker connections thus requires additional information, the nature of which is an open question. An important implication of these observations is that there are common building blocks/motifs and cortical network similarities between mammals from local to midrange distance scales (also indicated by strong adherence to EDR in this range), followed by species and/or individual dependent deviations at longer distances. One could then speculate that major aspects of diverse behavioral traits, including intelligence, are encoded in the long-range connectivity of the connectome (Wiesel, 1982).

## MATERIALS AND METHODS

### Data Preprocessing

In order to use the available input data, it needs to be organized in a format appropriate for the prediction algorithms. To generate the weights we compute the base-10 logarithm of all the nonzero entries of the FLN matrix (Markov et al., 2013b) (which range in order of magnitude from 10^−7^ to 1) then shift them by 7: *w*_*ij*_ = 7 + log_10_(*FLN*_*ij*_). The zero entries are left as zeroes. The resulting matrix has values between 0 and 7 (in both species) with 0 entries corresponding to nonlinks (i.e., nonconnected node pairs and elements on the main diagonal), the rest to actual links. The largest macaque distance is *D*_*max*_ = 58.2 mm and for mouse is 12 mm. For both species, the distance feature matrix ***D***_***f***_ = {31 · (*D*_*ij*_/*D*_*max*_)} with values ranging from 0 to 31^2^.

### Software Packages

For this work we used Python 3.7 and SciKit-Learn version 0.20.2. The computation of the ML and CL predictors, cross-validation, and analysis of the results were implemented in Python. General calculations and plotting functions are utilizing the standard packages of NumPy and Matplotlib.

#### Classical link predictor formulas.

Since we do not have incoming links except for injected areas, we need to modify slightly the predictor formulas as shown in Table 2.

**Table 2.** Classical, neighborhood-based link predictors for directed and weighted networks

**Method** (abbreviation)	**Formula**
Common neighbors v2 (CN2)	*CN*2(*u*, *v*) = $\frac{1}{2}\sum_{z\in I}$ [*w*(*z*, *u*) + *w*(*z*, *v*)]
Preferential attachment (PA2)	*PA*2(*u*, *v*) = $\left(\frac{\sum_{z\in \Gamma_{o}\left(u\right)}w\left(z,u\right)}{\left|\Gamma_{o}\left(u\right)\right|}\right)\left(\frac{\sum_{z\in \Gamma_{o}\left(v\right)}w\left(z,v\right)}{\left|\Gamma_{o}\left(v\right)\right|}\right)$
Adamic-Adar v2 (AA2)	*AA*2(*u*, *v*) = $\frac{1}{2}\left(\sum_{z\in I}\frac{w\left(z,u\right)+w\left(z,v\right)}{\log\sum_{x\in \Gamma_{o}\left(z\right)}w\left(x,z\right)}\right)$
Resource allocation v2 (RA2)	*RA*2(*u*, *v*) = $\sum_{z\in I}\frac{w\left(z,u\right)+w\left(z,v\right)}{\sum_{x\in \Gamma_{o}\left(z\right)}w\left(x,z\right)}$
Jaccard v2 (JA2)	*JA*2(*u*, *v*) = $\frac{\sum_{z\in I}\min\left(w\left(z,u\right),w\left(z,v\right)\right)}{\sum_{z\in I}\max\left(w\left(z,u\right),w\left(z,v\right)\right)}$

*Note*. The formulas have been adapted to be based on the out-link neighborhood information of the endpoints (***u***, ***v***) of the directed link to be predicted. Each formula provides a prediction score ***s***(***u***, ***v***) for that directed link. Here ***I*** denotes the set of all target (injected) areas and **Γ**_0_(***u***) denotes the neighbors of ***u***, including itself.

#### Machine learning classifiers and predictors.

All the classifiers used are implemented in the Python package scikit-learn; “defaults” refer to those parameters provided in version 0.20.2 of the library. We list the other parameters used for each classifier below.

- *K*-nearest neighbors (KNN): n_neighbors = 5, leaf = 30
- Decision tree (DT): defaults
- Random forest (RF): n_estimators = 200, criterion = 'gini'
- Multilayer perceptron (MLP): hidden layer size: 100, convergence error tolerance: 10^−6^, max iterations: 20
- Gradient boosting (GB): n_estimators = 100 (default), which is the number of boosting stages to perform. GB is robust to overfitting and larger values typically yield better performance. Max_depth = 7 (not default). This is the maximum depth of the individual regression estimators. It limits the number of vertices in the tree.
- AdaBoost (ADA): defaults
- Naïve Bayes (NBA): defaults

### Feature Vectors

Here we summarize the feature vectors that we used to train and test the classifiers. In each feature function in Table 3, the link in question is (*u*, *v*); *A* denotes the weight matrix; *D* denotes the distance matrix; *d*(*x*) denotes the outdegree of node *x* in *I*; and *I* denotes the set of injected areas (nodes) in the training set. Notice that the feature vectors have various lengths, as some provide more information than others.

**Table 3.** Machine learning feature functions used to train our classifiers

**Feature**	**Formula**
Weighted_common_neighbors (sum of FLN weights of links from source areas *u* and *v* to target *i*)	∑_*i*∈*I*_ [*A*(*i*, *u*) + *A*(*i*, *v*)]
Degree_plus_distance	{*d*(*u*), *d*(*v*), *D*(*u*, *v*)}
Adjacency	{*A*(*i*, *u*) > 0, *A*(*i*, *v*) > 0|∀*i* ∈ *I*}
Outdistance_source (vector of distances from source area *u* to the injected areas *I*)	{*D*(*i*, *u*)|∀*i* ∈ *I*}
Outdistance_target (vector of distances from the target area *v* to the injected areas *I*)	{*D*(*i*, *v*)|∀*i* ∈ *I*}
Outdistance (vector of distances from areas *u* and *v* to the injected areas *I*)	{*D*(*i*, *u*), *D*(*i*, *v*)|∀*i* ∈ *I*}
FLN	{*A*(*i*, *u*), *A*(*i*, *v*)|∀*i* ∈ *I*}
FLN_plus_distance	{*A*(*i*, *u*), *A*(*i*, *v*)|∀*i* ∈ *I*} ∪ {*D*(*u*, *v*)}

### Scaling and Leave-One-Out Analysis

We consider a random subset ℳ of *m* target areas, leave one target area out (of this *m*), then make the prediction for the out-links of the excluded area, based on the links of the remaining *m* − 1 areas in ℳ. We repeat this exclusion/prediction for every member of ℳ, obtaining a prediction error for each. These are then compared with the ground truth and the relative error computed, which we call internal relative error (internal to the selected subset ℳ). We then repeat this random selection of *m* subsets 500 times and average the internal errors.

## SUPPORTING INFORMATION

Supporting information for this article is available at https://doi.org/10.1162/netn_a_00345.

## AUTHOR CONTRIBUTIONS

Ferenc Molnár: Conceptualization; Formal analysis; Methodology; Software; Validation; Visualization; Writing – original draft; Writing – review & editing. Szabolcs Horvát: Data curation; Formal analysis; Validation; Visualization; Writing – review & editing. Ana Rita Ribeiro Gomes: Data curation; Formal analysis; Writing – review & editing. Jorge Martinez Armas: Formal analysis; Software; Validation; Visualization. Botond Molnár: Data curation; Formal analysis; Validation; Visualization. Mária Ercsey-Ravasz: Data curation; Formal analysis; Writing – review & editing. Kenneth Knoblauch: Data curation; Resources; Validation; Writing – review & editing. Henry Kennedy: Conceptualization; Data curation; Funding acquisition; Investigation; Project administration; Resources; Supervision; Writing – review & editing. Zoltan Toroczkai: Conceptualization; Formal analysis; Funding acquisition; Investigation; Methodology; Project administration; Supervision; Writing – original draft; Writing – review & editing.

## FUNDING INFORMATION

Zoltan Toroczkai, Directorate for Computer and Information Science and Engineering (https://dx.doi.org/10.13039/100000083), Award ID: IIS-1724297. Henry Kennedy, Agence Nationale de la Recherche (https://dx.doi.org/10.13039/501100001665), Award ID: A2P2MC ANR-17-NEUC-0004. Henry Kennedy, Agence Nationale de la Recherche (https://dx.doi.org/10.13039/501100001665), Award ID: ANR-17-FLAG-ERA-HBP-CORTICITY. Kenneth Knoblauch, Agence Nationale de la Recherche (https://dx.doi.org/10.13039/501100001665), Award ID: ANR-19-CE37–0025-DUAL_STREAMS. Maria Ercsey-Ravasz, Ministry of Education and Research, Romania (https://dx.doi.org/10.13039/501100006730), Award ID: CNCS-UEFISCDI. Maria Ercsey-Ravasz, FLAG-ERA, Award ID: COFUND-FLAGERA 2-CORTICTY. Maria Ercsey-Ravasz, FLAG-ERA, Award ID: COFUND-FLAGERA-ModelDXConsciousness. Maria Ercsey-Ravasz, ERA-NET, Award ID: ERANET-NEURON-2-UnscrAMBLY. Maria Ercsey-Ravasz, Ministerul Cercetării, Inovării şi Digitalizării (https://dx.doi.org/10.13039/100018987), Award ID: PN-III-P4-PCE-2021-0408. Botond Molnar, Universitatea Babeş-Bolyai (https://dx.doi.org/10.13039/501100006347), Award ID: SRG-UBB 32977/2023.

## Notes

^1^ The training of ML predictors may be sensitive to the order in which the training data is supplied.^2^ This value gives a good resolution on the distance range, but other similar values can also be used.

## TECHNICAL TERMS

Retrograde tract-tracing: An experimental technique that identifies the cell bodies of neurons whose terminals (synapses) are located in a region injected with a liquid tracer (Fast Blue or Diamino Yellow). The tracer is taken up through the synaptic cleft, transported along the pre synaptic neuron’s axon, labeling its cell body. It maps single-step, physical connectivity. Injected functional areas are called targets, the areas housing the labeled neurons are called sources.
Fraction of labeled neurons: The proportion of labeled neurons of a source area computed as the ratio between the number of labels found in that source divided by the total number of labels found in all the sources.
Edge-complete subgraph: It is a subgraph spanned by a set of nodes for which the status of connectivity between any two of them is fully known (including edge weight information).
Exponential distance rule: It expresses the experimental observation that the probability of a neuron creating connections at a distance, decays exponentially with the distance.
Imputation: Predicting the missing values from a data set based on information learned from the dataset.
Overfitting: When the parameters of a model are tuned such as to obtain the best fit for the training set but performs weakly on the test set. This happens, for example, because the model tries to fit the noisy part of the training set.
Classifiers: The task of predicting the category or class of an object, i.e., “classification”, using its attributes.
Modeling bias: Appears when an inadequate model, or working hypothesis is used to represent the data, e.g., using a linear function to fit a parabolic curve.
Feature vector: It is a vector that stores the variables or data based on which predictions are made by the method.
Supervised classifiers/regressors: Supervised classification or regression are machine learning approaches where a model is trained using labeled data (the labels form the ground truth). Typically, it works by finding the model’s parameters that minimize a given error function.
*k*-fold cross-validation: It is a random resampling procedure that separates the same dataset into testing and training sets, evaluating the model on each split, repeating the procedure, then reporting the statistics of the performance on all the splits used.
MAE: The mean absolute error between the predicted quantity and its ground truth value.
RMAE: Relative mean absolute error is the MAE divided by the ground-truth value.
Regression: The task of predicting the numerical value of a dependent variable using information on some independent variables.
