# Supplementary material for: Predictability of cortico-cortical connections in the mammalian brain
Source: Netw Neurosci. 2024 Apr 1;8(1):138–57. doi: 10.1162/netn_a_00345 (PMC10861169; doi:10.1162/netn_a_00345)
Supplement: Supplementary file 1 [file netn-8-1-138-s001.pdf]

Supplemental Material for

Predictability of cortico-cortical connections in the mammalian brain

Ferenc Molnár, Szabolcs Horvát, Ana R. Ribeiro Gomes, Jorge Martinez Armas, Botond Molnár Maria Ercsey-Ravasz, Kenneth Knoblauch, Henry Kennedy, Zoltan Toroczkai*

*Corresponding author: Zoltan Toroczkai
Email: toro@nd.edu

This PDF file includes:

Figures S1 to S14
Tables S1 to S3
SI References

Supplemental data files (downloadable, see descriptions in this file):

- `all_weighted_macaque_data.csv`
- `all_weighted_mouse_data.csv`
- `distances_macaque.csv`
- `distances_mouse.csv`
- `Macaque_29x91_Arithmean_DBV23.45_GB_FIN_unnormalized.csv`
- `Mouse_Database_GB_FIN_unnormalized.csv`
- `Macaque_29x91_Arithmean_DBV23.45_RF_FIN_unnormalized.csv`
- `Mouse_Database_RF_FIN_unnormalized.csv`

Supplementary Figures

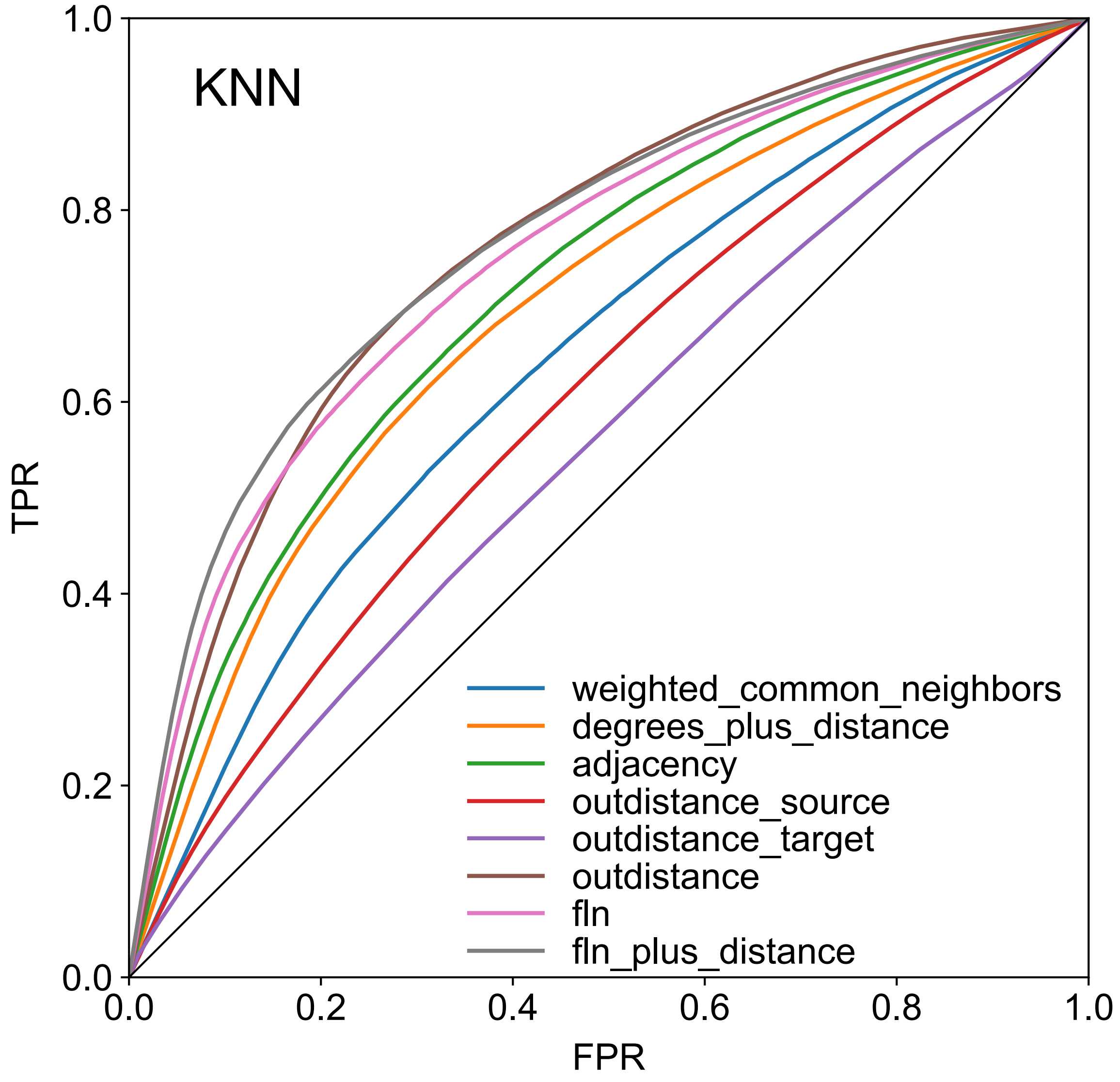

Fig. S1 ROC curves for binary link prediction with various ML features in the macaque, using the KNN classifier. Using 3-fold cross-validation, averaged over 100 samples.

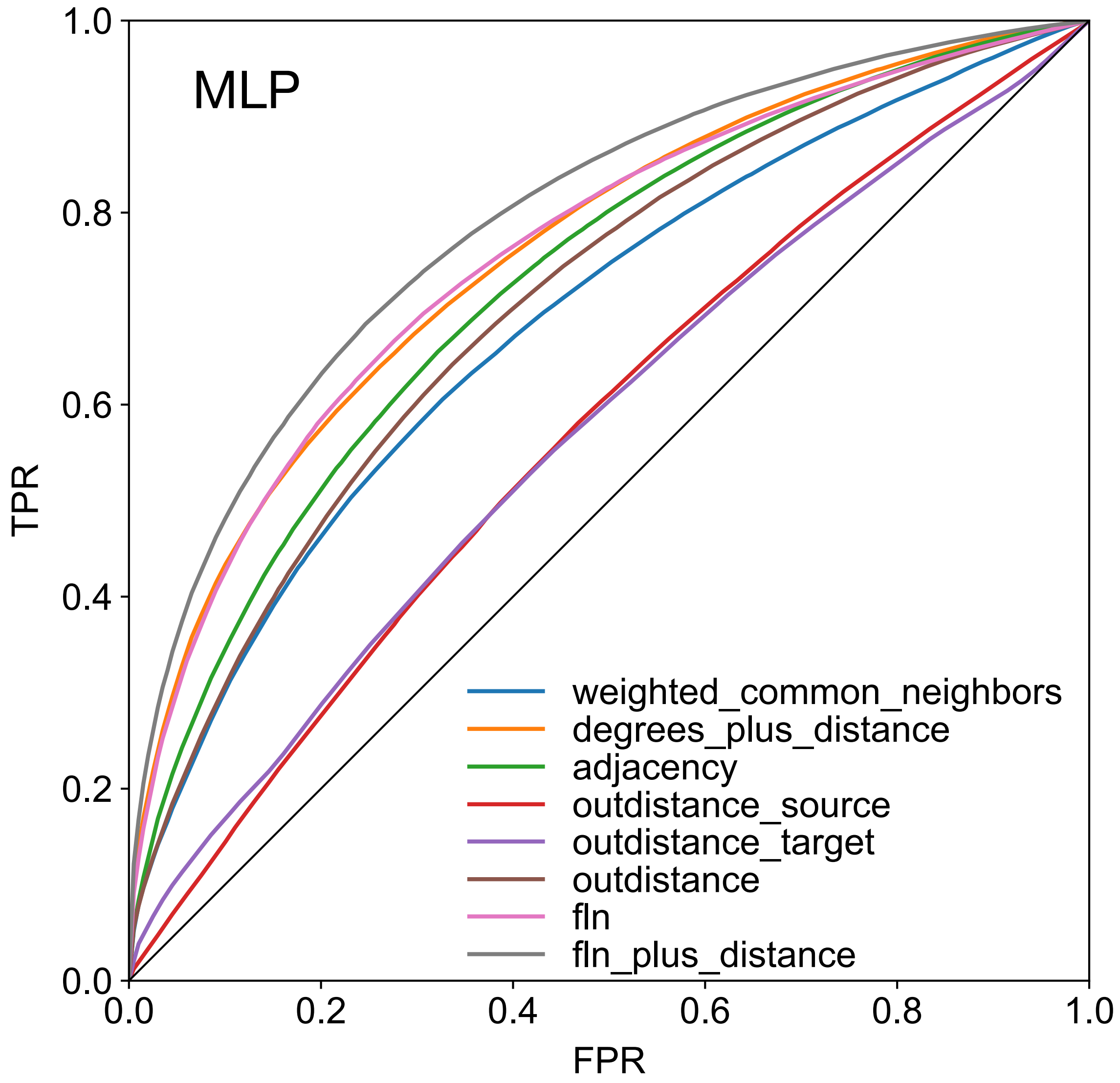

Fig. S2 ROC curves for binary link prediction with various ML features in the macaque, using the MLP classifier. Using 3-fold cross-validation, averaged over 100 samples.

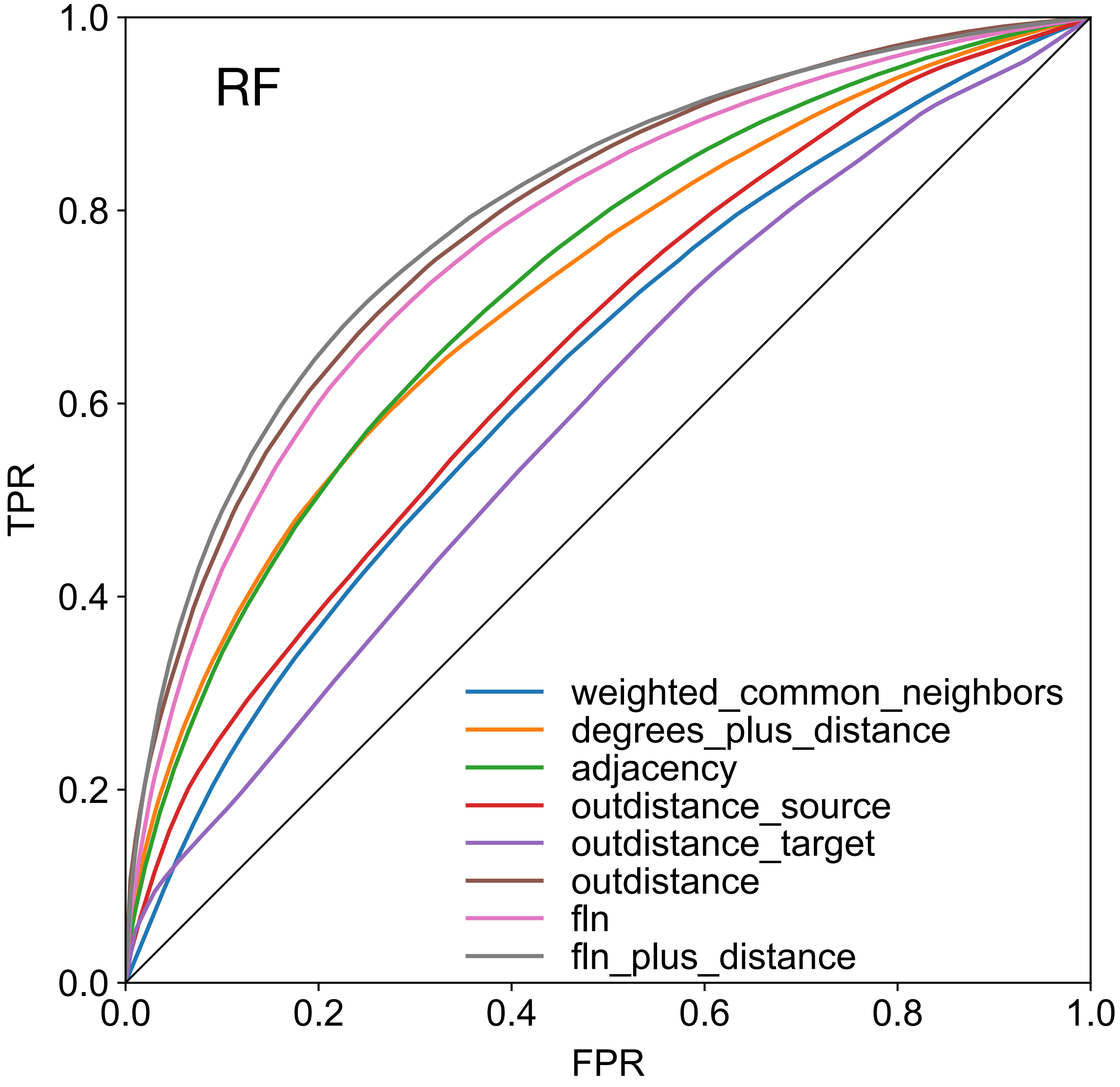

Fig. S3 ROC curves for binary link prediction with various ML features in the macaque, using the RF classifier. Using 3-fold cross-validation, averaged over 100 samples.

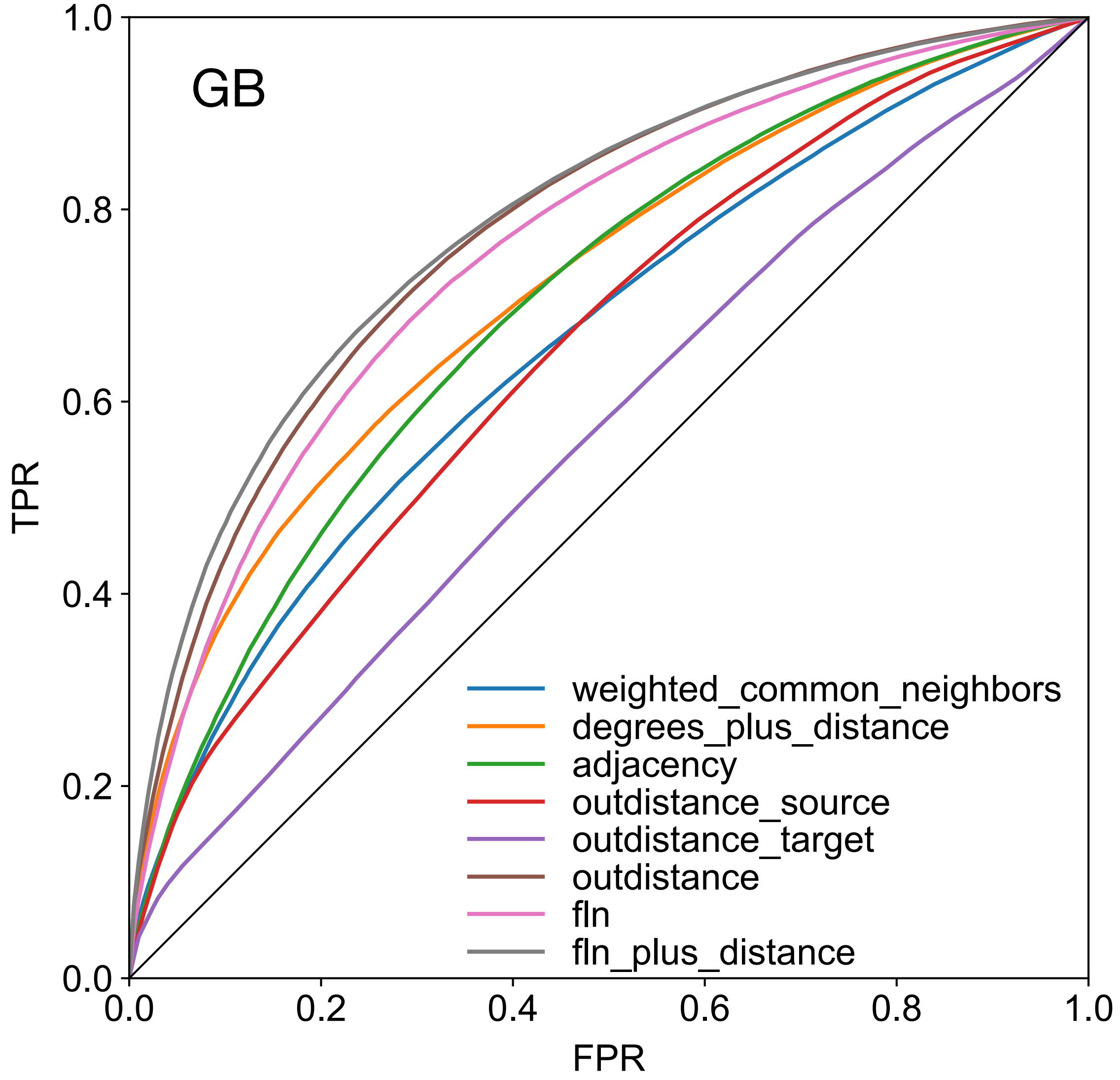

Fig. S4 ROC curves for binary link prediction with various ML features in the macaque, using the GB classifier. Using 3-fold cross-validation, averaged over 100 samples.

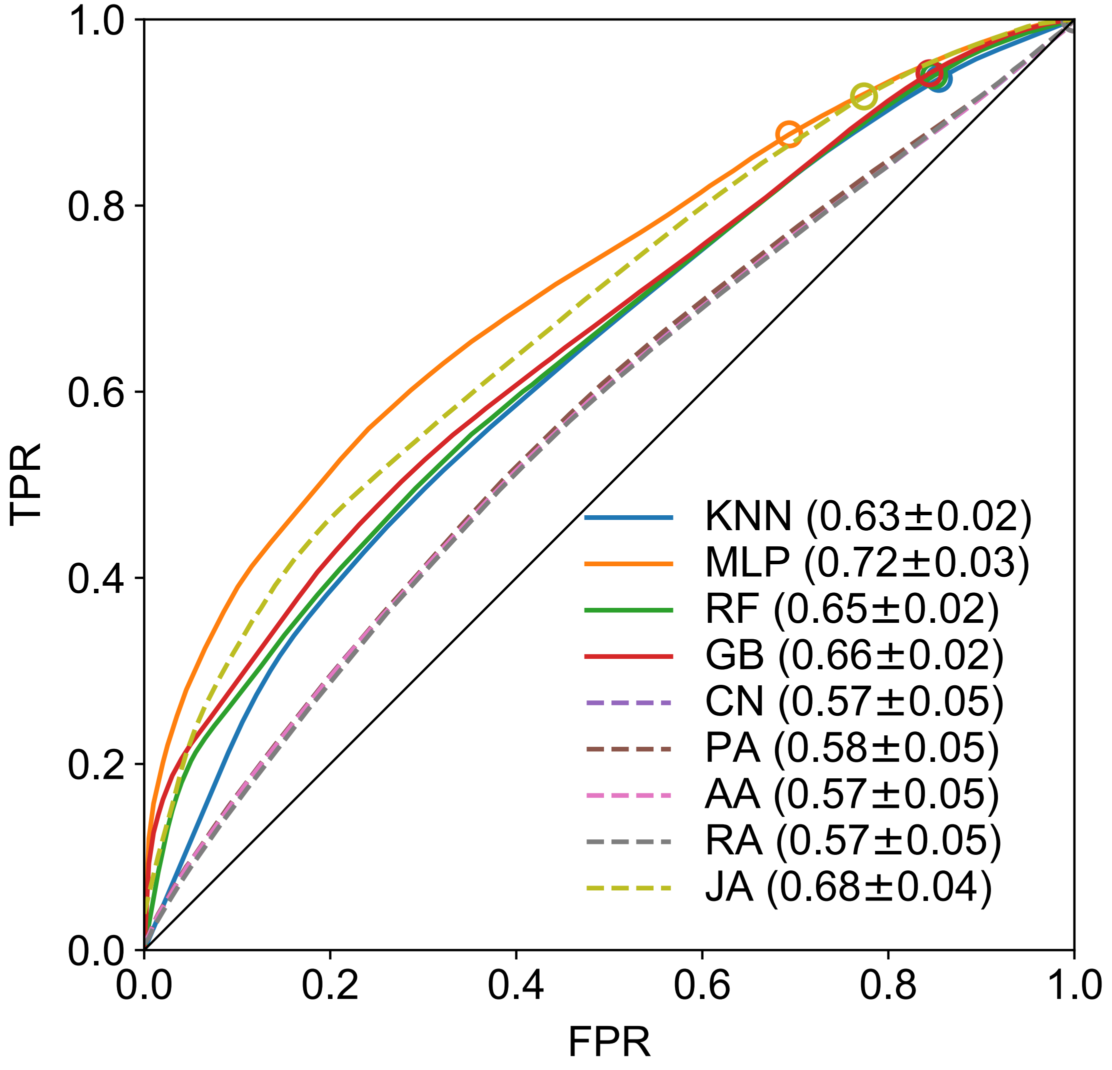

Fig. S5 ROC curves for binary link prediction in the macaque, using only the distance feature and both ML and CL algorithms. 3-fold cross-validation, averaged over 100 samples.

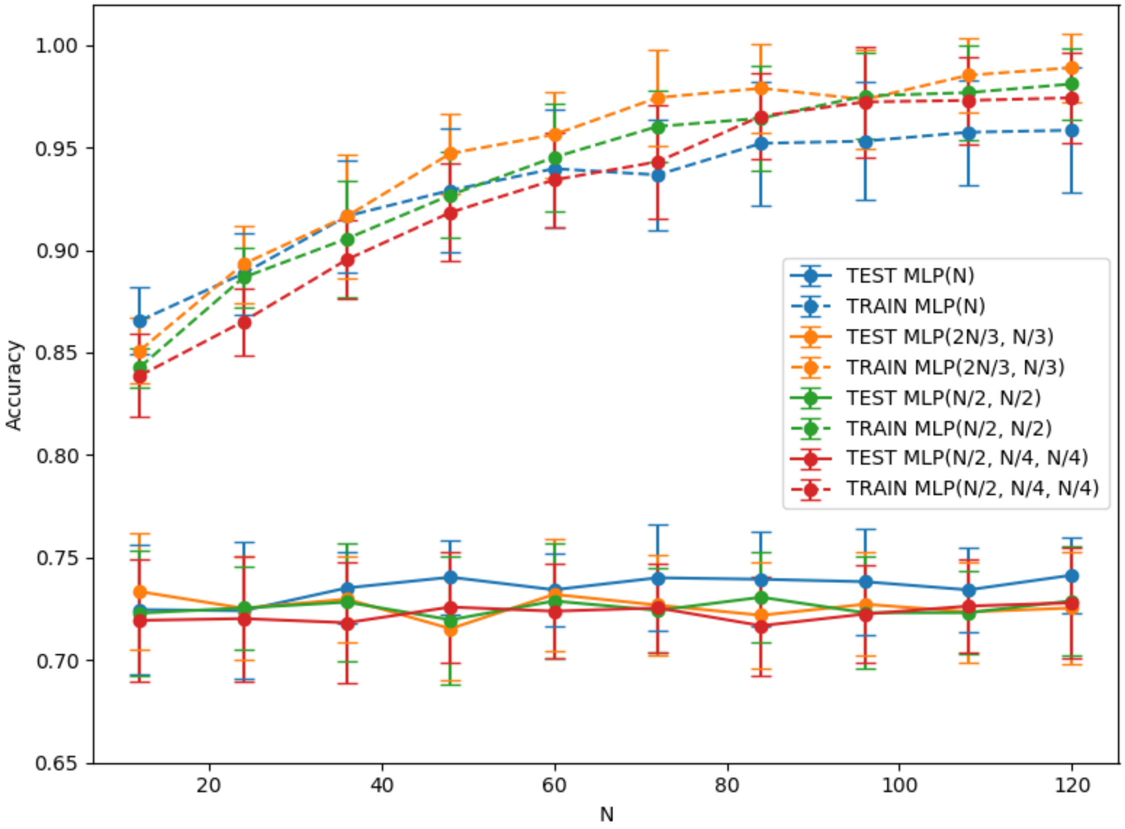

Fig. S6 Testing overfitting of the MLP predictor against the number of nodes in the hidden layer based on the macaque dataset. Accuracy on both the training set (dashed lines) and on the test set (continuous lines) as function of the hidden layer neuron count N, and as function of the organization of the hidden neurons into 1 (blue), 2 (orange and green) and 3 (red) layers. One can see that the internal organization does not significantly affect the performance, with the single layer doing slightly better than the multi-layer structure. This is a known fact in the machine learning community, namely, that true deep layered networks are not needed for small datasets.

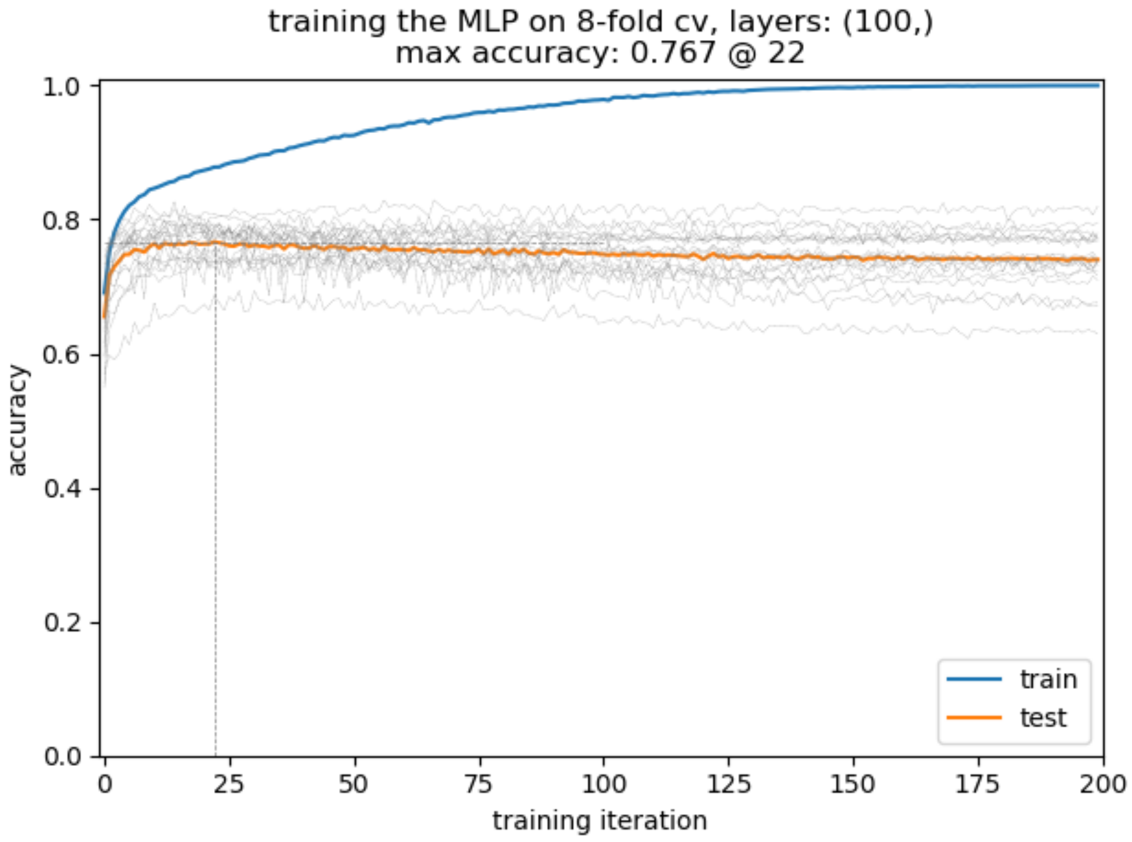

Fig. S7 Testing overfitting of the MLP predictor against the number of training epochs, macaque dataset. Another key test to do for testing overfitting is to test against the number of training epochs. Repeatedly training the network on the same data too many times also starts fitting the noise. This can be well seen in this figure. The gray curves indicate the test set accuracy of individual training runs; the orange curve shows their average. It shows that going beyond about 20 training epochs we start losing performance on the test set, while of course continuing to increase accuracy on the training set, because we are training to noise after that point. Thus, we choose 20 training epochs, to avoid this sort of overfitting.

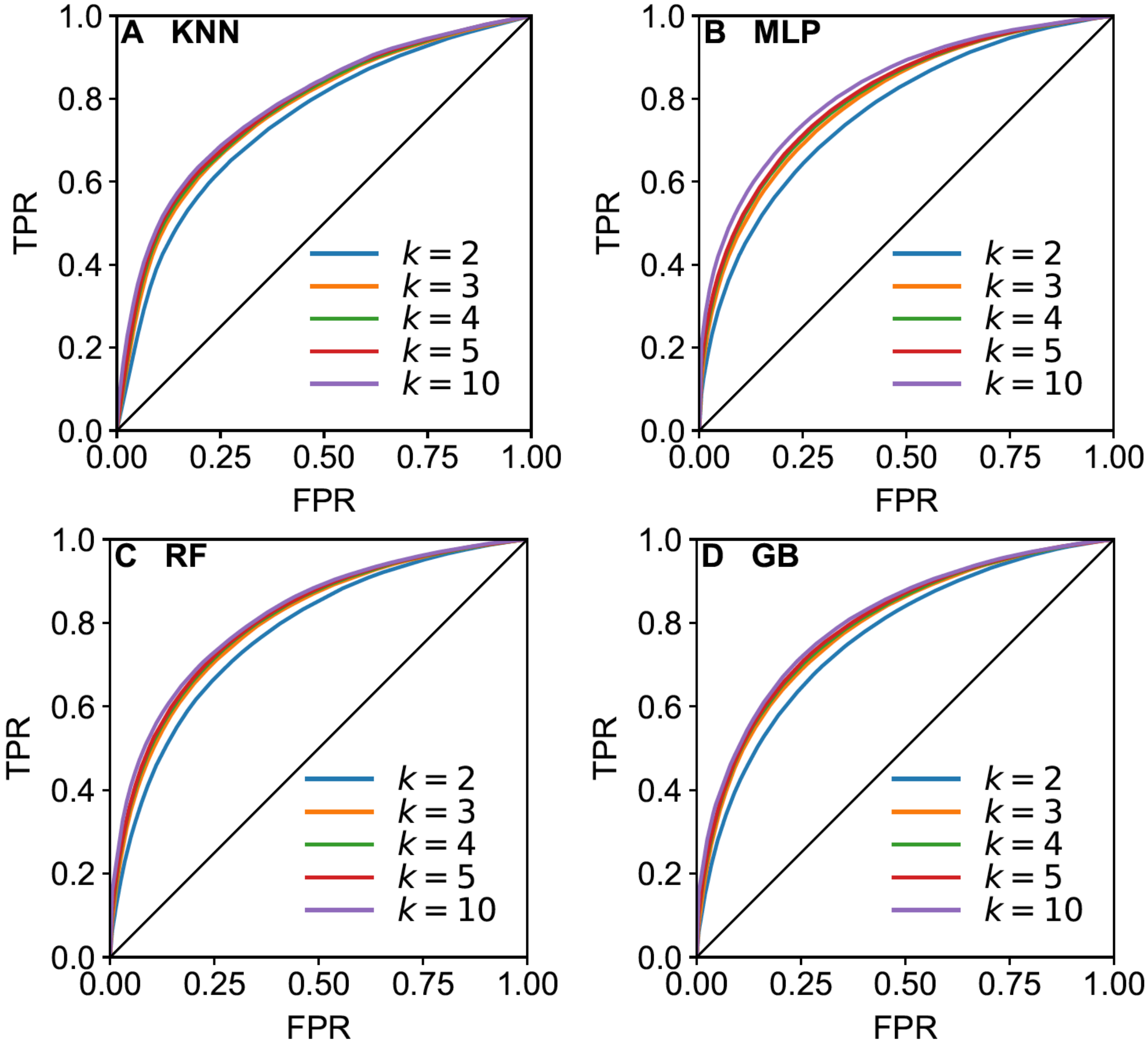

Fig. S8 ROC curves for binary link prediction using various fold sizes in k-fold cross-validation. As one can see, $\boldsymbol{k} = \mathbf{3}$ is already sufficient for achieving a close-to-the-best performance. Selecting higher $\boldsymbol{k}$ value, however, decreases the size of the dataset within a fold and increases uncertainty. Throughout we choose to work with $\boldsymbol{k} = \mathbf{3}$. Using the fln-plus-distance feature, averaged over 100 samples.

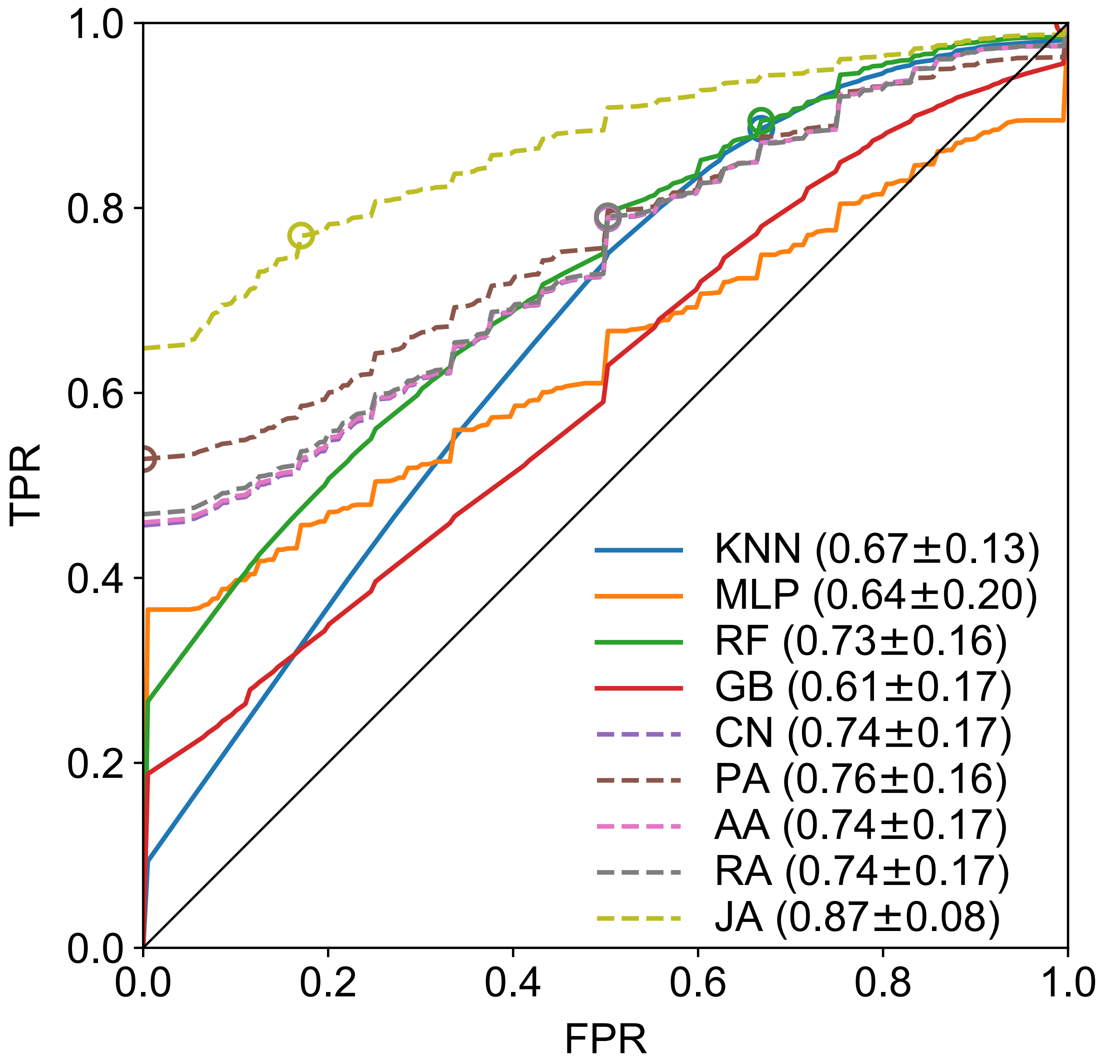

Fig. S9 ROC curves for binary link prediction in the mouse. These are based on ML (continuous lines) and CL (dashed lines) algorithms. 3-fold cross-validation, averaged over 100 samples.

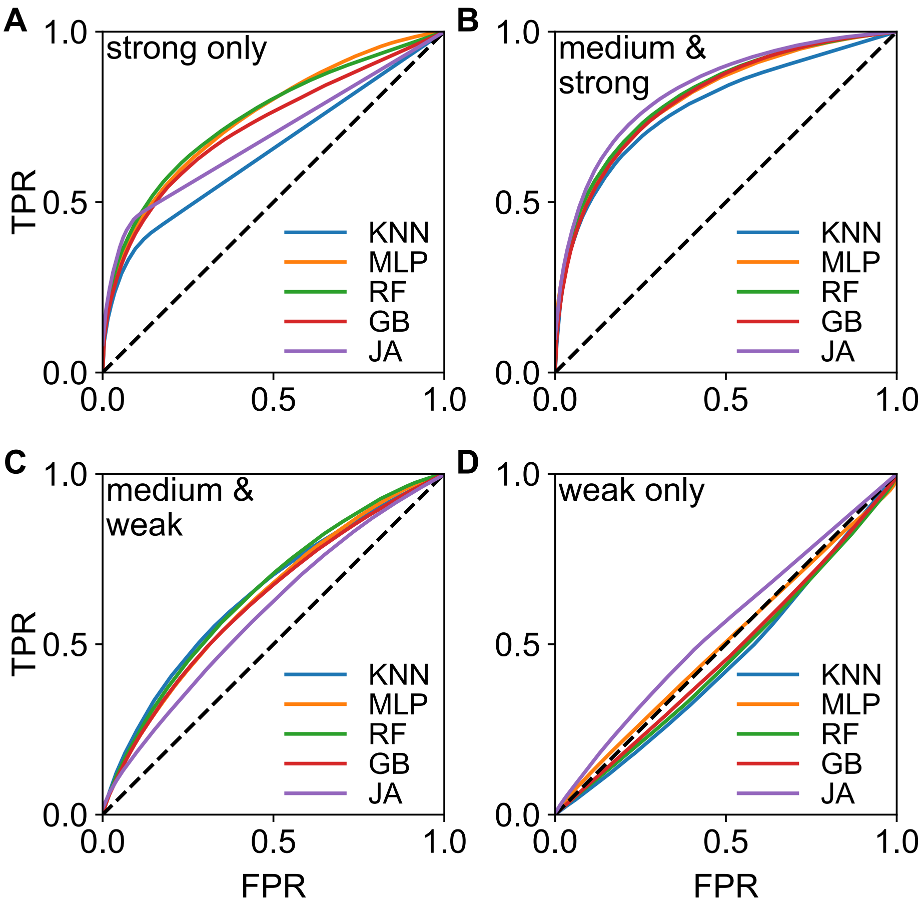

Fig. S10 ROC curves for binary link prediction after learning only within one weight class. The training set for each panel has been filtered to contain only links within that weight class (see main text for the definition of weight classes), before training. We then use the trained models to predict the existence of links in the test *set irrespective of their weight class*.

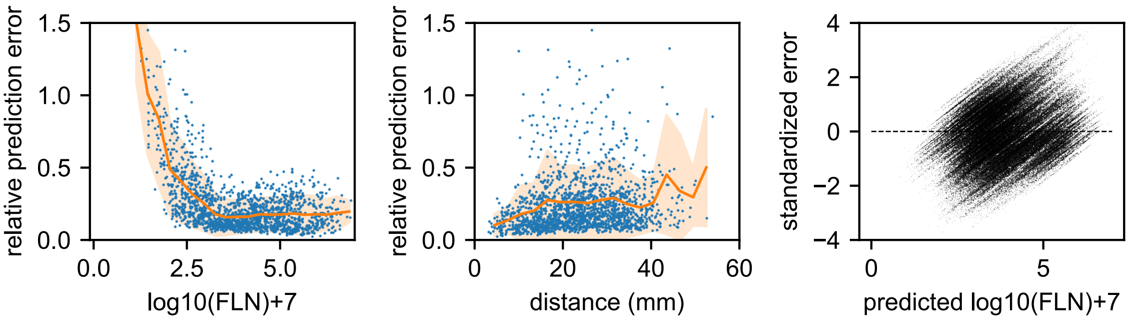

Fig. S11 Residuals analysis for the Gradient Boosting (GB) algorithm in cross-validation predictions. Macaque dataset. Based on the fln-plus-distance feature, 3-folded cross-validation, 100 samples. Residual: $Y_{true}(i) - Y_{pred}(i)$, or prediction error, for link i. Relative prediction error is $\left|Y_{true}(i) - Y_{pred}(i)\right|/Y_{true}(i)$ where $Y_{true}(i)$ is the true link weight in the data. Predictions are done on the dataset with no-links excluded (so all $Y_{true}(i)$ values are non-zero). Standardized error = $\left(Y_{true}(i) - Y_{pred}(i)\right)/std\_dev$. (A), (B) relative prediction errors (relative absolute residuals) as function of true weights and as function of distance. (C) shows the standardized residuals as function of predicted values. If the points scatter roughly symmetrically around zero along the y-axis, the model is good, most of the pattern/signal has been learned from the data.

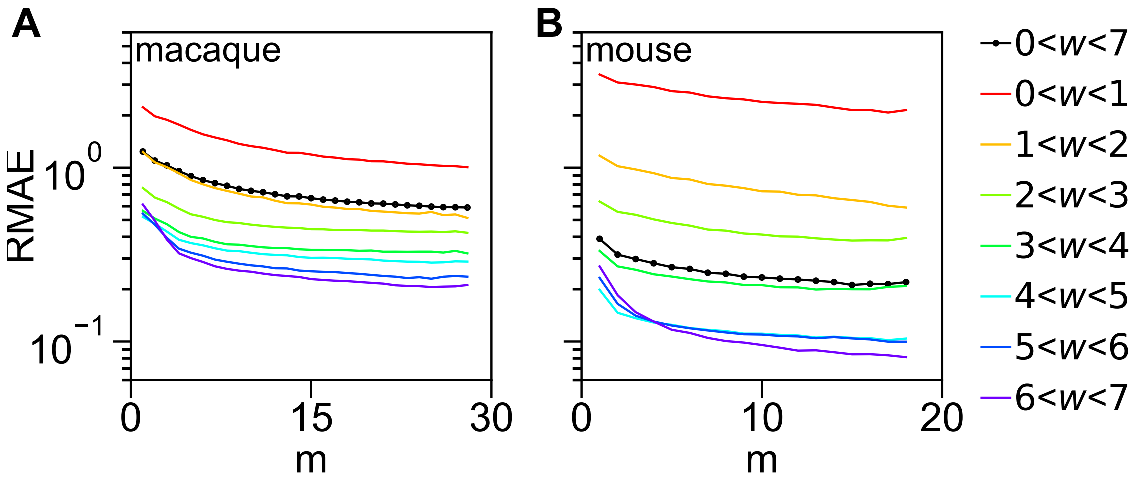

Fig. S12 Scaling of the external prediction errors. Here we consider a random subset of m areas from all the targets and instead of leaving an area out of the selected m (as for the case of internal errors shown in Figure 7 of the main text) we train the predictors based on all the data from the m targets to make a prediction for the out-links of *all the others*, *complementary* to m in the total set of targets (i.e., $29 - m$ for the macaque and $19 - m$ for the mouse). These predictions are compared to ground truth and the errors averaged over 500 random target set selections in the same way. We call these the *external relative prediction errors*. Although they look similar to the internal errors shown in Figure 7 of the main text, the values are somewhat different.

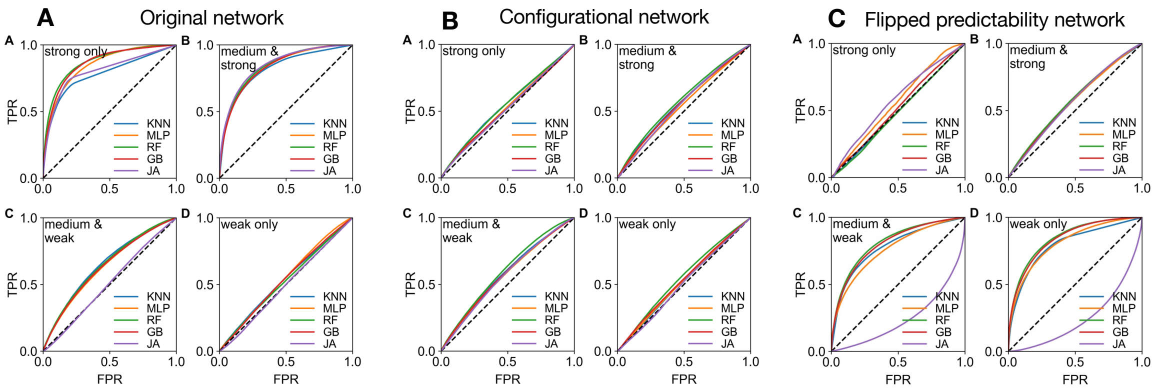

Fig. S13. ROC curves by link weight classes in macaque. (A) Is Fig 4 of the main text. (B) Shows the performance of the algorithms on the randomly rewired network, called the configurational model. (C) An example for a weighted network in which the predictability by weight class is flipped: now the weak and medium-weak links are more predictable. In (C) the Jaccard CL algorithm consistently makes the worst predictions because the rule in which it is based on (it is a pre-conceived model based predictor) is no longer valid for this (artificial) distribution of link weights.

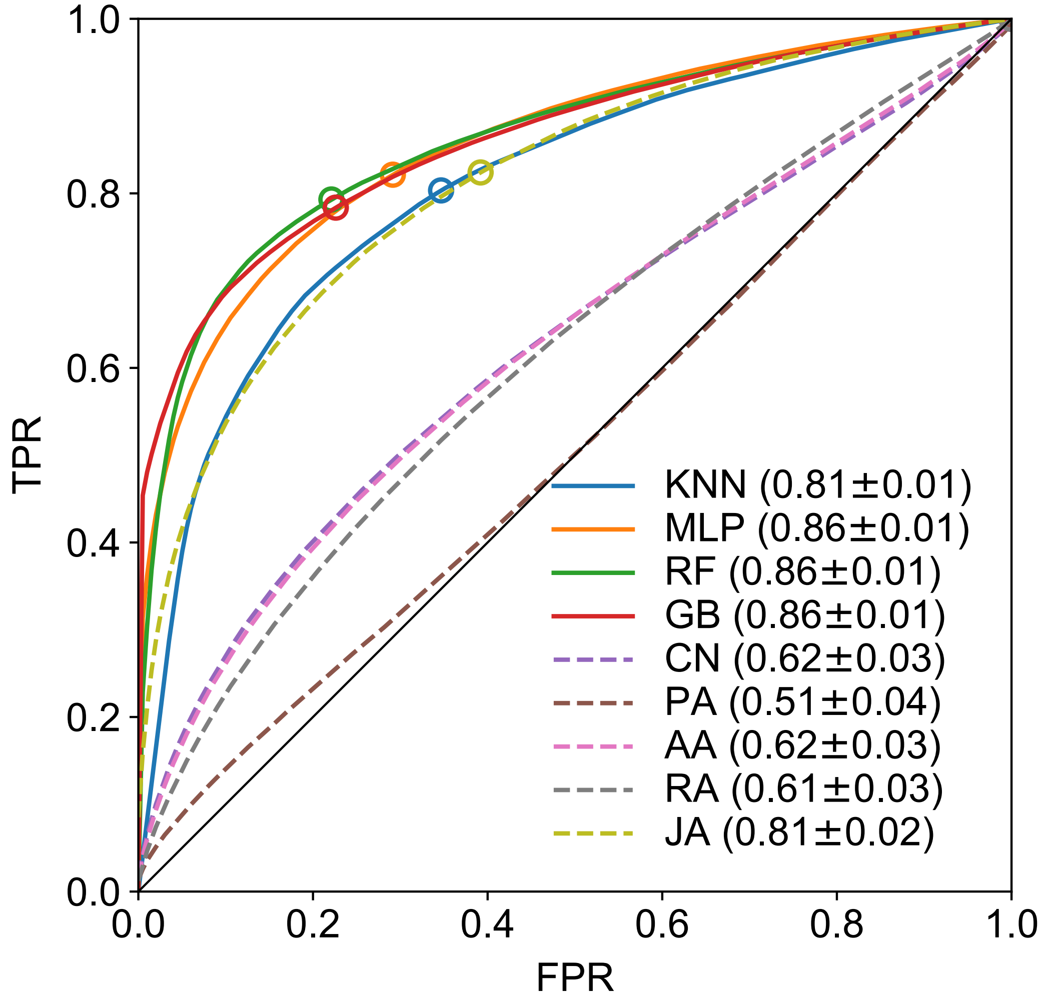

Fig. S14 ROC curves for binary link prediction in EDR networks. These are based on the distance matrix of the macaque, and $\lambda = 0.19\, mm^{-1}$ (Ercsey-Ravasz et al., 2013). Using 3-fold cross validation, averaged over 100 samples.

Supplementary Tables

Table S1. Abbreviations, area names and region assignments for the macaque.

Abbreviation	Area name	Region
1	Somatosensory area 1	Parietal
10	Area 10	Prefrontal
11	Area 11	Prefrontal
12	Area 12	Prefrontal
13	Area 13	Prefrontal
14	Area 14	Prefrontal
2	Somatosensory area 2	Parietal
23	Area 23	Cingulate
24a	Area 24, part a	Cingulate
24b	Area 24, part b	Cingulate
24c	Area 24, part c	Cingulate
24d	Area 24, part d	Cingulate
25	Area 25	Cingulate
29/30	Areas 29 and 30 of the retrosplenial cortex	Cingulate
3	Somatosensory area 3 (includes the primary somatosensory cortex)	Parietal
31	Area 31	Cingulate
32	Area 32	Cingulate
35/36	Areas 35 and 36 of the perirhinal cortex	Temporal
44	Area 44	Prefrontal
45A	Area 45A	Prefrontal
45B	Area 45B	Prefrontal
46d	Area 46, dorsal part	Prefrontal
46v	Area 46, ventral part	Prefrontal
5	Somatosensory area 5	Parietal
7A	Area 7A	Parietal
7B	Area 7B	Parietal
7m	Area 7m	Parietal
7op	Area 7op	Parietal
8B	Area 8B	Prefrontal
8l	Area 8l	Prefrontal
8m	Area 8m	Prefrontal
8r	Area 8r	Prefrontal
9	Area 9	Prefrontal
9/46d	Area 9/46d	Prefrontal
9/46v	Area 9/46v	Prefrontal
AIP	Anterior intraparietal area	Parietal
Core	Auditory core (includes the primary auditory cortex)	Temporal
DP	Dorsal prelunate area	Parietal
Ento	Entorhinal cortex	Temporal
F1	Frontal area F1 (primary motor cortex)	Frontal
F2	Frontal area F2	Frontal
F3	Frontal area F3	Frontal
F4	Frontal area F4	Frontal
F5	Frontal area F5	Frontal
F6	Frontal area F6	Frontal
F7	Frontal area F7	Frontal
FST	Fundus of the superior temporal sulcus	Temporal

Gu	Gustatory cortex	Frontal
Ins	Insular cortex	Parietal
IPa	Intraparietal sulcus associated area in the superior temporal sulcus	Temporal
LB	Belt region of the auditory cortex, lateral part	Temporal
LIP	Lateral intraparietal area	Parietal
MB	Belt region of the auditory cortex, medial part	Temporal
MIP	Medial intraparietal area	Parietal
MST	Medial superior temporal area	Temporal
MT	Middle temporal area	Temporal
OPAl	Orbital periallocortex	Prefrontal
OPro	Orbital proisocortex	Prefrontal
Pi	Parainsular cortex	Parietal
PBc	Parabelt region of the auditory cortex, caudal part	Temporal
PBr	Parabelt region of the auditory cortex, rostral part	Temporal
PGa	PG associated area of the superior temporal sulcus	Temporal
PIP	Posterior intaparietal area	Parietal
Pir	Piriform cortex	Temporal
ProM	Area ProM (promotor)	Frontal
ProSt	Prostriata	Temporal
SII	Secondary somatosensory area	Parietal
STPc	Superior temporal polysensory area, caudal part	Temporal
STPi	Superior temporal polysensory area, intermediate part	Temporal
STPr	Superior temporal polysensory area, rostral part	Temporal
Sub	Subicular complex	Temporal
TEad	Anterior TE, dorsal part	Temporal
TEa/m a	Superior temporal sulcus ventral bank area, anterior part	Temporal
TEa/m p	Superior temporal sulcus ventral bank area, posterior part	Temporal
TEav	Anterior TE, ventral part	Temporal
TEO	Temporal area TE, occipital part	Occipital
TEOm	Temporal area TE, occipitomedial part	Temporal
TEpd	Posterior TE, dorsal part	Temporal
TEpv	Posterior TE, ventral part	Temporal
TH/TF	Areas TH and TF of the parahippocampal cortex	Temporal
Temp.Pole	Temporal pole	Temporal
TPt	Temporoparietal area	Temporal
V1	Visual area 1 (primary visual cortex)	Occipital
V2	Visual area 2	Occipital
V3	Visual area 3	Occipital
V3A	Visual area 3, part A	Occipital
V4	Visual area 4	Occipital
V4t	Visual area 4, transitional part	Temporal
V6	Visual area 6	Parietal
V6A	Visual area 6A	Parietal
VIP	Ventral intraparietal sulcal area	Parietal

Table S2. Abbreviations, area names and region assignments for the mouse.

Abbreviation	Area name	Region
A	Anterior area	Occipital
ACAd	Anterior cingulate area, dorsal part	Cingulate
ACAv	Anterior cingulate area, ventral part	Cingulate
AId	Agranular insular area, dorsal part	Insular
AIp	Agranular insular area, posterior part	Insular
AIv	Agranular insular area, ventral part	Insular
AL	Anterolateral area	Occipital
AM	Anteromedial area	Occipital
AUDd	Auditory cortex, dorsal area	Temporal
AUDp	Auditory cortex, primary area	Temporal
AUDpo	Auditory cortex, posterior area	Temporal
AUDv	Auditory cortex, ventral area	Temporal
DP	Dorsal posterior area (also known as PD)	Temporal
ECT	Ectorhinal area (also referred to as area 36)	Temporal
FRP	Frontal pole	Frontal
GU	Gustatory area	Insular
ILA	Infralimbic area	Frontal
LI	Laterointermediate area	Occipital
LLA	Laterolateral anterior area	Occipital
LM	Lateromedial area	Occipital
MM	Mediomedial area	Cingulate
MOp	Motor cortex primary	Frontal
MOs	Motor cortex secondary	Frontal
ORBl	Orbitofrontal area, lateral part	Frontal
ORBm	Orbitofrontal area, medial part	Frontal
P	Posterior area	Occipital
PERI	Perirhinal area (also referred to as area 35)	Temporal
PL	Prelimbic area	Frontal
PM	Posteromedial area	Occipital
POR	Postrhinal area	Occipital
PORa	Postrhinal anterior	Occipital
RL	Rostrolateral area	Occipital
RSPagl	Retrosplenial area, agranular part	Cingulate
RSPd	Retrosplenial area, dorsal part	Cingulate
RSPv	Retrosplenial area, ventral part	Cingulate
SSp-bfd	Somatosensory cortex primary, barrel field	Parietal
SSp-lj	Somatosensory cortex primary, lower jaw	Parietal
SSp-ll	Somatosensory cortex primary, lower limb	Parietal
SSp-nm	Somatosensory cortex primary, nose and mouth	Parietal
SSp-tr	Somatosensory cortex primary, trunk	Parietal
SSp-ul	Somatosensory cortex primary, upper limb	Parietal
SSp-un	Somatosensory cortex primary (unassigned)	Parietal
SSs	Somatosensory cortex, secondary	Parietal
TEa	Temporal area, anterior part	Temporal
TEp	Temporal area, posterior part	Temporal
V1	Primary visual area	Occipital
VISC	Visceral area	Insular

Table S3. Prediction errors by link weight based only on existing links (w > 0). Definitions are the same as in Table 1 of the main text. Since the non-links are excluded from the data, the predictors can only predict actual links (cannot predict non-links). Notice, the errors in general are somewhat smaller than in the case when we include the non-links as well.

Non-links excluded	Macaque		Mouse		Mac/Mus
	MAE	RMAE	MAE	RMAE	RMAE ratio
Weak ($\boldsymbol{w_{cut}} < \boldsymbol{w} < \mathbf{3}$)	0.970	0.439	1.029	0.449	0.977
Weak-&-Medium ($\boldsymbol{w_{cut}} < \boldsymbol{w} < \mathbf{5}$)	0.787	0.268	0.637	0.194	1.383
Medium-&-Strong ($\boldsymbol{w} > \mathbf{3}$)	0.783	0.174	0.555	0.124	1.404
Strong ($\boldsymbol{w} > \mathbf{5}$)	1.005	0.178	0.562	0.101	1.769
All links ($\boldsymbol{w} > \boldsymbol{w_{cut}}$)	0.835	0.248	0.613	0.164	1.511

Supplementary data files (downloadable)

`all_weighted_macaque_data.csv`:

Contains all weighted data for the macaque for 29 area injections, including the ground truth FLN_{ij}, $w_{ij} = 7 + \log_{10}(FLN_{ij})$ and predicted w_{ij}-s and corresponding relative mean absolute errors (RMAE).

`all_weighted_mouse_data.csv`:

Contains all weighted data for the mouse for 19 area injections, including the ground truth FLN_{ij}, $w_{ij} = 7 + \log_{10}(FLN_{ij})$ and predicted w_{ij}-s and corresponding relative mean absolute errors (RMAE).

`distances_macaque.csv`:

Contains all macaque interareal distances in mm-s (91×91 values).

`distances_mouse.csv`:

Contains all mouse interareal distances in mm-s (47×47 values).

Full Interareal Networks (FIN)

Starting from the 29x91 weighted data matrix for the macaque and the 19x47 data matrix for the mouse, weighted FINs were imputed both for macaque (91x91 matrix) and mouse (47x47 matrix) using GB and RF, respectively. Note, in the experimentally obtained data files (29x91 for macaque and 19x47 for mouse) the FLN values are normalized around every target (label counts in a source area divided by the sum of all label counts in all areas extrinsic to the target, for that target injection), because label counts in the target areas themselves are not available. Thus, in the data matrices (29x91 mac and 19x47 mus), all rows add up to one. However, this is not a hard constraint for the prediction algorithms and thus in the FINs the row sums are no longer unity. They can be normalized, if the reader wishes to do so, we are including the output of the prediction algorithms as they are generated.

`Macaque_29x91_Arithmean_DBV23.45_GB_FIN_unnormalized.csv`

macaque FIN imputed with GB.

`Mouse_Database_GB_FIN_unnormalized.csv`

mouse FIN imputed with GB.

`Macaque_29x91_Arithmean_DBV23.45_RF_FIN_unnormalized.csv`

macaque FIN imputed with RF.

`Mouse_Database_RF_FIN_unnormalized.csv`

mouse FIN imputed with GB.

SI References

Ercsey-Ravasz, M., Markov, N.T., Lamy, C., Van Essen, D.C., Knoblauch, K., Toroczkai, Z., and Kennedy, H. (2013). A predictive network model of cerebral cortical connectivity based on a distance rule. Neuron *80*, 184–197.
